# The mechanism of trans-δ-viniferin inhibiting the proliferation of lung cancer cells A549 by targeting the mitochondria

**DOI:** 10.3389/fphar.2023.1190127

**Published:** 2023-05-18

**Authors:** Ruochun Yin, Yiling Zhang, Liping Su, Dongdong Chen, Shidi Lou, Xuecai Luo, Lin Wang, Rupei Tang, Liang Zhang, Xiaohe Tian

**Affiliations:** ^1^ School of Life Science, Anhui University, Hefei, China; ^2^ Hefei Ting Xiandu Biological Technology Co, Ltd., Hefei, China; ^3^ Institutes of Physical Science and Information Technology, Anhui University, Hefei, China; ^4^ National Engineering Laboratory for Cereal Fermentation Technology, Jiangnan University, Wuxi, China; ^5^ Department of Radiology, West China Hospital of Sichuan University, Chengdu, China

**Keywords:** trans-δ-viniferin, lung cancer, A549, metabolome, proteome, mitochondria

## Abstract

Trans-δ-viniferin (TVN), as a natural extract, is a resveratrol dimer with attractive biological activities, particularly its anti-tumor character. However, the mechanism of TVN interfering with cancerous proliferation has not been fully understood. Herein in this study, we found that TVN could trigger cancerous mitochondrial membrane potential (ΔΨm) reduction, with intracellular reactive oxidative species (ROS) level increasing, leading to apoptosis, which makes TVN a promising candidate for lung cancer cells A549 treatment. Therefore, this study provides TVN as an option to meet the demand for higher antitumor availability with lower biotoxicity and other clinical applications.

## 1 Introduction

**FIGURE 1:**
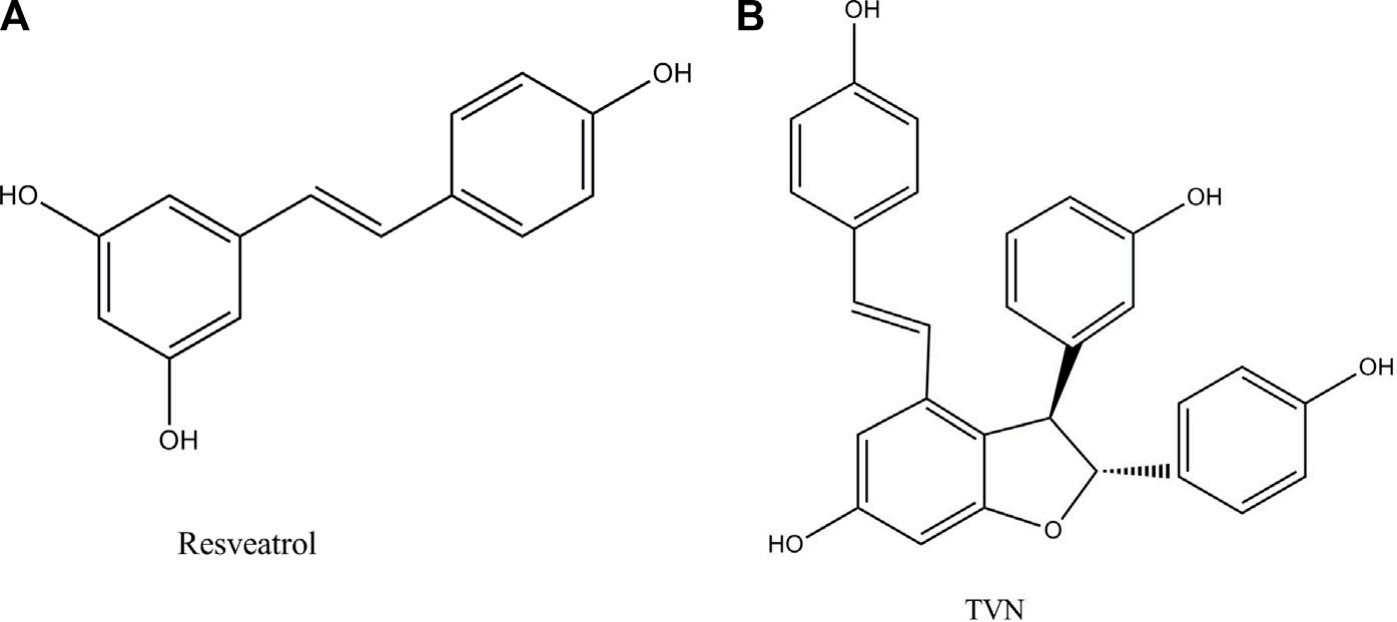
Chemical structures of resveratrol in this study and its dimer TVN.

Resveratrol (Figure 1A) is a small molecule of plant antitoxin produced by mechanical damage, ultraviolet radiation, or fungal infection (Bostanghadiri et al., 2017). Studies have shown that it has a variety of therapeutic effects such as anti-inflammatory (Poulsen et al., 2015), antioxidant (Chikara et al., 2018), anti-tumor (Jiang et al., 2017) (human ovarian cancer cells (Lang et al., 2015), lung cancer (Yin et al., 2013), and breast cancer (Zhang et al., 2014)), and cardioprotection (Das et al., 1999). The study reported that lung cancer occurred frequently, and the therapeutic effect of lung cancer had not been significantly improved in recent years (Beadsmoore and Screaton, 2003).

Resveratrol oligomers are polyphenolic organic compounds, which are natural products synthesized by plants. Nowadays, the widespread reports that they can promote health and treat diseases, especially in the field of anti-tumor, have attracted more professional interest (Xue et al., 2014). TVN (Figure 1B), a resveratrol dehydrodimer, was firstly obtained through the biotrans-formation of resveratrol treated with horseradish peroxidase and H_2_O_2_ (Langcake and Pryce, 1977) and was also detected in grapes and red wine (Vitrac et al., 2005). However, few works of literature focused on TVN affecting lung cancer cell development. The efficiency and mechanism of TVN in the treatment of lung cancer should be complemented with a comprehensive assessment of TVN’s anti-tumor activities.

Mass spectrometry-based label-free quantification methods had previously been reported for proteomics examinations (Zhong et al., 2018). Compared with isotope labeling methods, this method has the advantages of simpler operation, larger dynamic range, and wider coverage (Wang et al., 2021). Herein, proteomics and metabolomics were subjected to evaluation to investigate the effect of TVN on the proliferation of A549 cells. We found TVN could inhibit the proliferation of A549 cells in a dose- and time-dependent manner. Further experiments showed that TVN induced ΔΨm reduction with ROS level increasing, leading to apoptosis. Metabolomics and proteomics evaluations confirmed that significantly different proteins and metabolites were mainly concerned with metabolic pathways such as the PI3K/Akt signaling pathway. Hence, we conjectured that TVN could induce apoptosis by downregulating A549 cell proliferation via a ROS/PI3K/Akt pathway. This may be convenient to comprehend the apoptosis mechanism of A549 cells initiated by TVN.

## 2 Materials and methods

### 2.1 Materials

Resveratrol (>98%) and other chemicals were purchased from Hefei Bomei Biological Technology Co., Ltd. TVN (purity ≥98%), synthesized by our laboratory, and its structure was identified by MS and NMR spectroscopies. Laccase was purchased from Sigma. Ammonium acetate (CH_3_COONH_4_), ammonium hydroxide (NH_4_OH), ammonium fluoride (NH_4_F), and formic acid (HCOOH) were purchased from Sigma Aldrich. Acetonitrile was purchased from Merck.

Human A549 cell lines and human Beas-2B cell lines were both purchased from the cell bank of the Chinese Academy of Sciences (Shanghai, China). The cells were grown in RPMI-1640 (Hyclone Laboratories Inc., Logan, UT, United States) and supplemented with 10% fetal bovine serum (Hyclone Laboratories Inc., Logan, UT, United States) and antibiotics (100 U/mL penicillin, 0.1 mg/mL streptomycin) (Hyclone Laboratories Inc., Logan, UT, United States). The medium was changed once every 48 h, and human A549 cells were cultured in a 37°C 、5% CO_2_ incubator.

### 2.2 Methods

#### 2.2.1 Cell proliferation assay

Cells were first seeded into a 96-well plate at a density of 5*10^4^ cells per well and incubated overnight. Then the cells were treated with TVN at different concentrations for various hours and 0.1‰ dimethyl sulfoxide (DMSO) was employed as vehicle control. Then we added 3-(4,5-dimethylthiazolyl-2)-2,5-diphenyltetrazolium bromide (MTT) to each well at a final concentration of 0.5 mg/ml. After 4 h, we put the Jiazan crystal into 130 μ Dissolve in L dimethyl sulfoxide and measured its absorbance at 490 nm.

#### 2.2.2 Colony formation assay

A total number of 200 cells were cultured in Petri dishes (60 mm) containing 5 mL medium (10 μM TVN or 1‰ DMSO), which was maintained for about 10 days at 37°C in an incubator with 5% CO_2_. Then, we added 3.5% paraformaldehyde for fixation, removed the fixative 15 min later, washed the cells slowly with running water, and then counted after 20 min of crystal violet staining. The assay was repeated three times with duplicate samples.

#### 2.2.3 Cell morphological study

Hoechst33342/PI double staining was conducted to examine the apoptotic rate of A549 cells. In brief, A549 cells were cultured in a medium supplemented with or without TVN onto the glass bottom cell culture dishes. After 60 h of treatment, the cells were washed twice with phosphate buffer (PBS) and stained with Hoechst 33342 and PI fluorescence in a dark environment at 4°C for 10 min. Then the cells were washed twice with PBS and were immediately observed under a confocal laser scanning microscope.

#### 2.2.4 Cell apoptosis assay

The analysis of apoptosis was performed according to the manufacturer’s instructions for the Annexin V-FITC/PI apoptosis kit (BestBio China). A549 cells were seeded in 6-well plates (BestBio China) with 3×10^5^ cells per well and treated with 10 μM TVN or 1‰ DMSO for 60 h. Then, the cells were harvested and washed twice with cold PBS and resuspended in 400 µL of binding buffer containing 5 µL of Annexin V-FITC and 10 μL of PI working solution at 4°C for 15 min in the dark. Apoptosis was analyzed by flow cytometry (BD Biosciences company, United States of America) for at least 10,000 events.

#### 2.2.5 Flow cytometric measurement of intracellular ROS levels

Intracellular ROS levels were measured by flow cytometry. After treatment with 10 μM TVN or 1‰ DMSO for 60 h, cells were washed with PBS, and DCFH-DA was added, with a final concentration of 2 nM/tube. The samples were analyzed by flow cytometry (BD Biosciences company, United States) for at least 10,000 events.

#### 2.2.6 Measurement of mitochondrial membrane potential (ΔΨm)

A Mitochondrial Membrane Potential Assay Kit was used to measure the mitochondrial membrane potential according to the operating instructions in the manual. Briefly, 3*10^5^ cells were collected by trypsinization and incubated with JC-1 for 15 min at 37 °C in the dark. The stained cells were washed with PBS twice and 500 μL of working solution was added to each well. Then the cells were immediately analyzed by flow cytometry (BD Biosciences company, United States of America) for at least 10,000 events. JC-1 aggregates in the polarized mitochondrial matrix form J-aggregates, which emit red fluorescence at 595 nm when excited at 525 nm.

#### 2.2.7 LC-MS/MS analysis

A UHPLC (1290 Infinity LC, Agilent Technologies) coupled to a quadrupole time-of-flight (AB Sciex Triple TOF 6600) in Shanghai Applied Protein Technology Co., Ltd. was used to analyze the data.

Samples were analyzed using a 2.1 mm × 100 mm ACQUIY UPLC BEH 1.7 µm column (waters, Ireland) for HILIC separation. A (25 mM ammonium acetate and 25 mM ammonium hydroxide in water) and B (acetonitrile) form a mobile phase in ESI-positive and negative modes. The gradient was 85% B for 1 min and was linearly reduced to 65% in 11 min, and then was reduced to 40% in 0.1 min and kept for 4 min, and then was increased to 85% in 0.1 min, with 5 min re-equilibration period employed.

Detailed information about cell materials and treatments, mass spectrometry-based label-free quantitative proteomics methods, and untargeted metabolomics methods are described in Supplementary Materials.

Proteomics raw data is available free of charge via the Internet at http://www.iprox.org/ and the project ID is IPX0001973001.

## 3 Results

### 3.1 Cytology experiment

**FIGURE 2:**
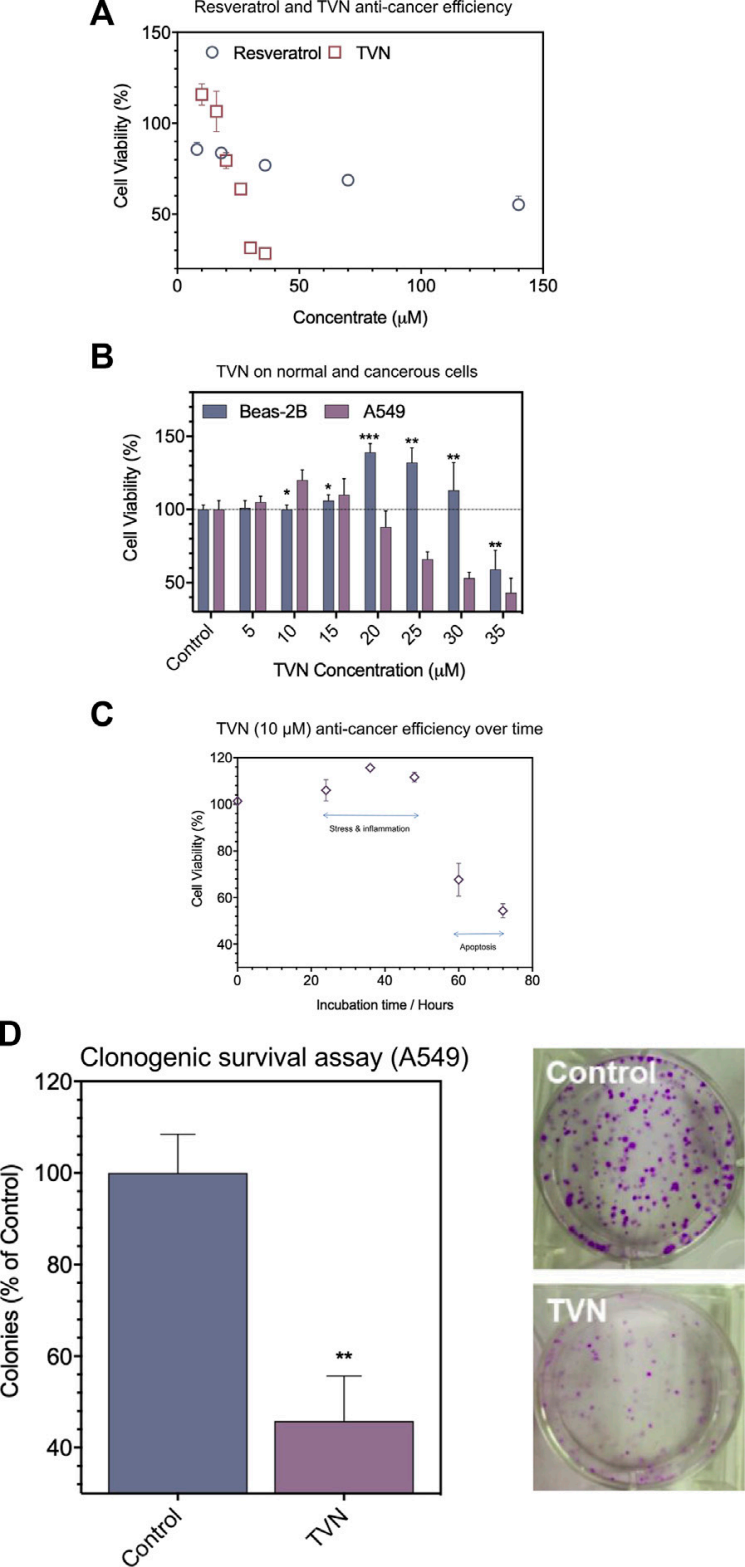
**(A)** The effect of resveratrol and TVN on the viability of A549 cells was assessed by the MTT assay; **(B)** The effect of TVN on the viability of Beas-2B cells and A549 cells was assessed by the MTT assay; **(C)** The time-dependent effect of TVN on the viability of A549 cells was detected by the MTT assay; **(D)** Clonogenic survival assay showed that the number of A549 cells colonies decreased with TVN. The results are shown relative to non-treated cells (control), defined as 100%. Data were expressed as mean ± S.D. (n = 3); *p < 0.05; **p < 0.01; ***p < 0.0001 compared with control.

Firstly, TVN was synthesized and catalyzed by horseradish peroxidase and confirmed by UHPLC and MS analysis (Zhang et al., 2017a) (Supplementary Figure S1). The biocompatibility of resveratrol and TVN was evaluated in living cells by detecting the effects on the proliferation of A549 cancer cells using classic MTT assay (Figure 2A). The results showed that both resveratrol and TVN could inhibit the growth of A549 cells, while TVN suggested much higher inhibition rare It was also clearly observed that, up to a certain concentration range of TVN (10 μM–15 μM), as shown in Figure 2B, at TVN concentrations of 10 and 15 micro rubs, the cell viability of A549 cells was even higher than the control cells, indicating potential cell stress which could boost cell proliferation (Battistelliet al., 2016). Above 15 μM concentrations (20–35 μM) were proven to be cytotoxic toward A549 cancer cells in a dose-dependent manner. Based on the MTT data, we calculated IC50 values of 175 μM and 27.36 μM, respectively, and the ability of TVN to inhibit A549 cell growth was 6.40 times higher than that of resveratrol.

To examine if TVN displayed different impacts on cancerous cells and normal cells, Beas-2B cells (human normal lung epithelial cells) were treated in parallel with TVN. Figure 2B shows that the viability of Beas-2B cells had not been much influenced in the range of 5–10 μM concentrations with mild cell stress. Meantime, however, A549 cells demonstrated an apparent decrease as a function of concentration. Subsequently, we used 10 μM concentration to carry out the follow-up experiment and indicated the cancerous cell viability could significantly alter within 72 h (Figure 2C), and possibly due to programmed cell death (e.g., apoptosis). Subsequently, a colony formation assay was developed to evaluate the effect of TVN on the proliferation of A549 cancer cells. The number of A549 cells treated was significantly reduced compared to the control group (Figure 2D). These data indicated the positive effect of TVN to some extent.

**FIGURE 3:**
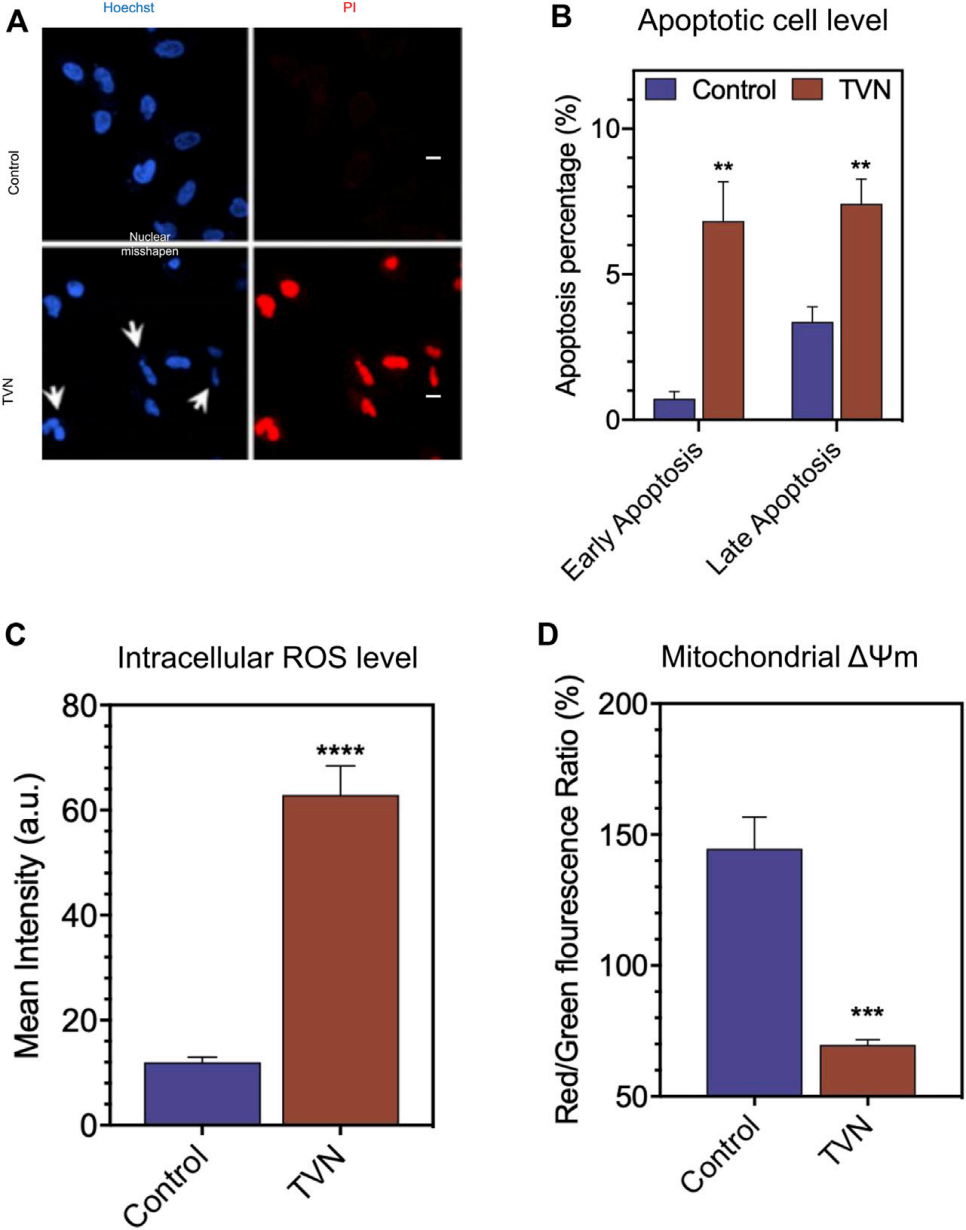
**(A)** The morphological aspects of the A549 cells. The apoptosis of A549 cells was detected by Hoechst33342/PI fluorescent double staining kit and then examined by Laser scanning confocal microscope (scale bar: 20 μM); **(B)** The apoptosis was detected by FCM Annexin V/PI staining; **(C)** The measurement of intracellular ROS production in A549 cells was done using the fluorescent probe DCFH-DA via FlowJo 7.6 analysis; **(D)** The ΔΨm was detected by FCM via JC-1 staining. The data represented the mean ± SD of three independent experiments. *p < 0.05, **p < 0.01, ***p < 0.001, and ****p < 0.0001, compared with the control.

Next, we examined the physiological status of the cells by Hoechst33342/PI (Propidium Iodide) double staining. Typically, PI can stain cells that lose membrane integrity in late apoptosis and present red fluorescence, while Hoechst33342 can label both living and dead cells. Figure 3A shows that the nuclear morphology of the control group was normal, and the blue fluorescence color was evenly distributed; whereas, after TVN treatment, the nucleus of some cells contracted into crescent-shaped fragments, and the PI channels could see red fluorescence in the visual field, which was significantly higher than that of the control group. The above results strongly suggested that TVN could induce apoptosis in A549 cells. In order to further verify the ability of TVN to induce apoptosis in A549 cancer cells, Annexin V-FITC/PI double staining and flow cytometry were conducted. After treatment with TVN (10 μM) for 60 h, the number of viable cells was reduced, whereas the number of apoptotic cells was increased, especially early apoptosis (Figure 3B, Supplementary Figure S2A). These data indicated that TVN may inhibit the proliferation of A549 cancer cells through an apoptosis pathway.

It is well known that ROS is an inevitable product of cell metabolism. High concentrations of ROS can directly or indirectly participate in cell signal transduction and induce apoptosis within cells, which is a common pathogenesis of aging diseases including tumors, diabetes, and Alzheimer’s disease (Wick et al., 2000; Petrosillo et al., 2004). To investigate the oxidative damage effect of TVN on A549 cells, a ROS assay was performed to measure the intracellular ROS level. DCFH-DA can be hydrolyzed by intracellular esterases, whereas ROS oxidates non-fluorescent DCFH-DA to convert to the highly fluorescent 2′,7′-dichlorofluorescein (DCF). As shown in Figure 3C and Supplementary Figure S2B, the intracellular ROS level of TVN-incubated cells was significantly elevated, compared to the non-treated cells. It was reported that most energy and ROS *in vivo* generate in the mitochondria, which is the most sensitive organelle to ROS impact. ROS causes oxidative damage by oxidizing mitochondrial cardiolipin, mitochondrial DNA, and proteins important to mitochondria and then induces cell apoptosis (Wang et al., 2014). Furthermore, mitochondria dysfunction is often related to the changes in ΔΨm (Joo et al., 2016). To evaluate whether mitochondrial membrane integrity was damaged by treatment with TVN and ΔΨm, A549 cells were examined using JC-1 fluorescent probe that stains mitochondria in living cells in a membrane potential-dependent manner (Figure 3D, Supplementary Figure S2C). Cells treated with TVN showed a reduction in Red/Green fluorescence intensity, which indicated that TVN possibly induced apoptosis in A549 cells through the mitochondria-related pathway. The ATP synthesis rate is positively correlated with the electrochemical H+ gradient on both sides of the mitochondrial inner membrane. TVN reduces ATP synthesis by reducing mitochondrial membrane potential. The decrease of ΔΨm activated the synthesis of enzymes related to energy metabolism, promoted the release of ATP, and inevitably increased intracellular ROS, which led to the subsequent changes in the metabolic flow (Liu et al., 2009). The absorption of electrons by TVN would also improve the intracellular redox potential (Soden and Dobson, 2003). It was hypothesized that TVN may induce apoptosis through ROS-mediated mitochondria-related pathways.

### 3.2 Identification of metabolites

In order to further explore the mechanism, we also used label-free proteomics and non-target metabolomics to analyze the differential in protein and metabolites of cells that were treated with or without TVN. As shown in Supplementary Figure S3, the total ion current chromatograms of metabolites were detected by using a solvent system. Therefore, there were significant differences in the relative intensity and peak values between the positive mode (Supplementary Figure S3A) and the negative mode (Supplementary Figure S3B). Nevertheless, the difference between QCs samples was very insignificant, suggesting that variation remained in the optimal range.

**FIGURE 4:**
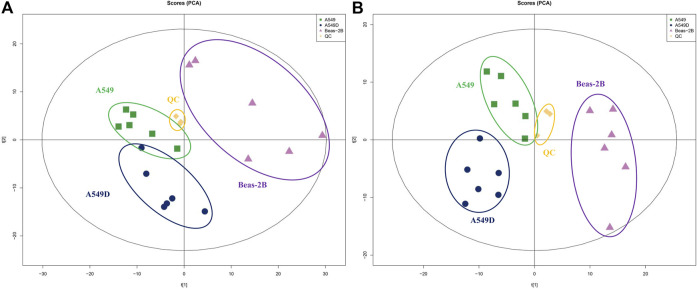
PCA analysis of metabolites by the solvent system under positive **(A)** and negative **(B)** ion modes. Spots in green show samples from the A549 group; spots in navy blue indicate samples from the A549D group; spots in purple indicate samples from the Beas-2B group; spots in yellow indicate samples from the QC group.

In addition, we conducted a PCA analysis to briefly discuss metabolic changes. The PCA data showed clearly separated clusters, indicating obvious separations between A549 and A549D (A549 + TVN) or Beas-2B groups. All quality control (QC) samples were collected, and their dispersion was significantly lower than that of experimental samples, indicating that the stability of the system was satisfactory and the differences between groups were real (Zhan et al., 2018) (Figure 4). The clustering of different groups had been realized which displayed that TVN had an effective intervention effect on the physiological metabolic status of A549 cells.

In addition, OPLS-DA analysis, serviced as a supervised method for pattern recognition, was performed on the data in the comparison of A549 *versus* Beas-2B and A549D *versus* A549. As shown in Supplementary Figure S4, groups positive for Beas-2B, A549, and A549D were separated in the OPLS-DA score plots (Supplementary Figures S4A, S4E). For metabolites detected in the negative mode, score plots for each comparison were also presented as separated clusters (Supplementary Figures S4C, S4G). OPLS-DA analysis implied that the explanatory power of the data and the predictive power of the model are excellent. The results of permutation tests showed no overfitting of data (Supplementary Figures S4B, S4D, S4F, S4H), indicating the model of OPLS-DA was valid (Zhang et al., 2017b).

### 3.3 Analysis of different metabolites

A large number of metabolites in the comparison of A549 *versus* Beas-2B and A549D *versus* A549 were identified in the positive and negative modes, respectively. According to literature reports, proline metabolism involves the mutual conversion of proline and glutamic acid, which is related to the reduced ability of proline in the cytoplasm. In mitochondria, the proline cycle affects electron chain transmission and regulates reduction status, ATP production, and reactive oxygen species production by producing NADH and FADH_2_ (Zheng et al., 2021). It can be seen from Supplementary Table S1 that the content of D-proline in A549 cells decreased to half after treatment with TVN, and the changing trend of glutamic acid content was similar to that of D-proline, suggesting that TVN could inhibit the metabolic activity of A549 cells. The analysis of significantly different metabolites of A549 *versus* Beas-2B is found in Supplementary Table S2.

**FIGURE 5:**
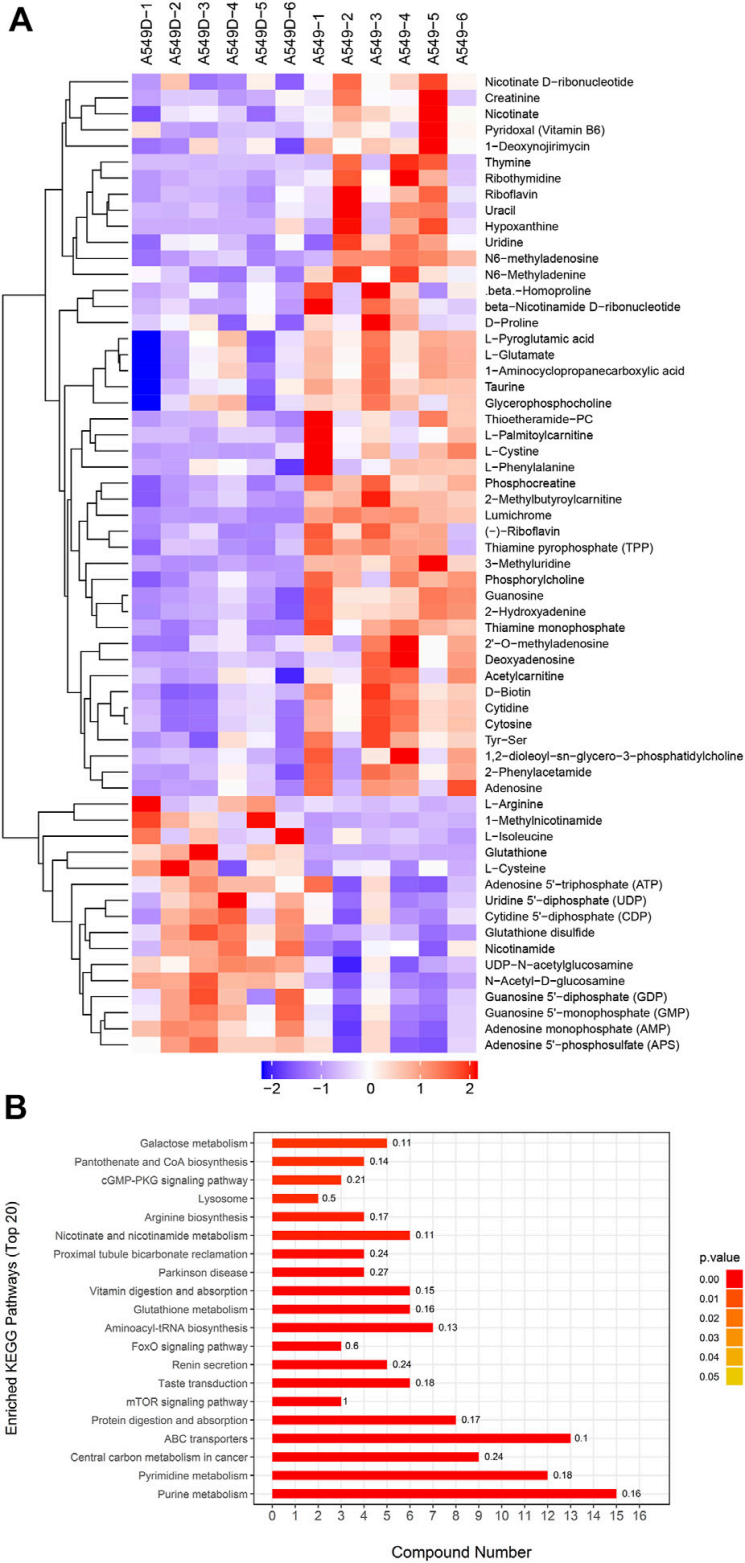
**(A)** The clustering results of hierarchical cluster analysis are based on the significantly different metabolites from A549D *versus* A549 under positive and negative modes; **(B)** Enriched KEGG pathway for the differential metabolites from A549D *versus* A549.

Based on OPLS-DA, a further refined set of metabolites was obtained using the criteria of VIP>1 and *p* < 0.05. In order to cluster the significantly different metabolites, a two-way hierarchical cluster analysis was performed in the comparison of A549D *versus* A549 (Figure 5A). The clustering results of the hierarchical cluster analysis of A549 *versus* Beas-2B are found in Supplementary Figure S5A. The clustering of the results demonstrated that the identified differential metabolites were reasonable and accurate. Besides, we also could see the content of the same metabolite between different samples and its changing trend.

### 3.4 Enrichment analysis of differential metabolites KEGG pathway

The differential expression metabolism from the A549D group *versus* the A549D group was analyzed by the Fisher accurate test (Figure 5B). The results showed that the important pathways such as the purine metabolism, pyrimidine metabolism, central carbon metabolism in cancer, ABC transporters, and mTOR signaling pathway changed significantly. The enrichment analysis of the differential metabolites KEGG pathway from the A549 group *versus* the Beas-2B group is found in Supplementary Figure S5B. According to these flow cytometry experiments and histological analysis, it was speculated that TVN was attracted to the low pH environment outside the inner membrane of the mitochondria, where it reduced ΔΨm until it was degraded by secondary metabolic enzymes.

### 3.5 Quantitative proteome analysis

According to the criteria of *p*-value<0.05 and fold changes>2.0, 551 proteins exhibited significant differential expression from A549 *versus* Beas-2B; In the Beas-2B group, there were 253 proteins with increased abundance and 298 proteins with decreased abundance (Supplementary Figure S6A).

Furthermore, 41 proteins with differential abundance were identified from A549D *versus* A549. Compared with the A549 group, there were 16 increased abundances and 25 reduced abundances in the A549D group (Supplementary Figure S6B).

### 3.6 Gene Ontology (GO) analysis of identified proteins

**FIGURE 6:**
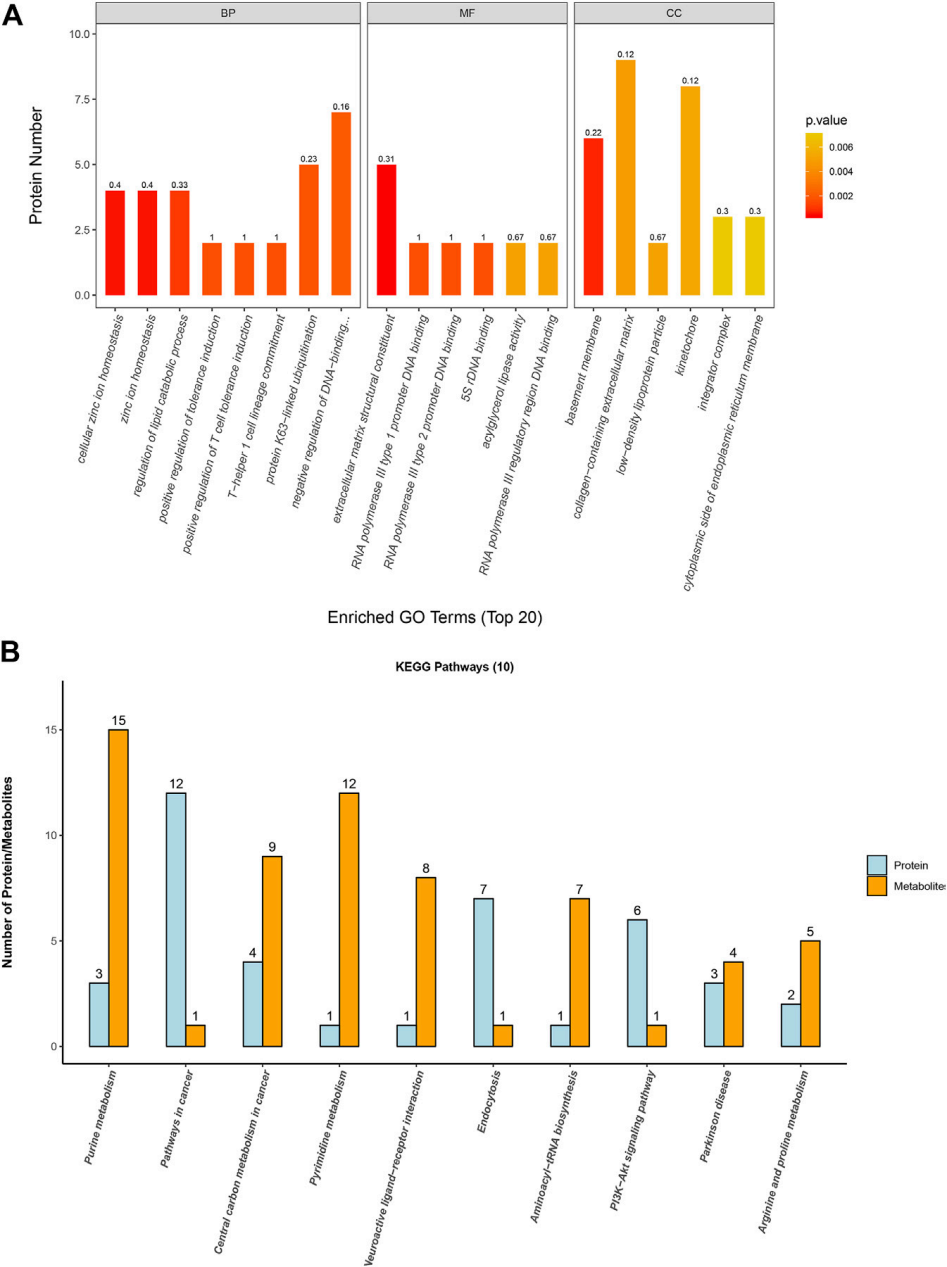
**(A)** Gene Ontology analysis for biological processes (BP), molecular functions (MF), and cellular components (CC) of identified proteins based on significant different proteins from the A549D group *versus* the A549 group; **(B)** The first 10 pathways that contained the highest number of proteins and metabolites.

The GO annotation of differentially expressed proteins was classified into biological processes (BP), cell components (CC), and molecular functions (MF). We determined the biological activity of proteins with significant differences (*p* < 0.05) based on MF, localized proteins based on CC, and participating biological pathways based on BP. Compared with the A549 group, there were differential proteins affected in the ion homeostasis, cell regulation, binding activity, and enzyme activity in the A549D group (Figure 6A). Like MF, enzymes related to catalytic activity include cytochrome c oxidase subunit 3, dehydrogenase/reductase SDR family member 4, and inosine-5-monophosphate dehydrogenase 1. Glutathione reductase (GR) is an important component of the glutathione (GSH) antioxidant system, which can clear ROS and repair oxidative damage (Samarghandian et al., 2017). GR catalyzes the reduction of glutathione disulfide (GSSG) to glutathione using reduced nicotinamide adenine dinucleotide phosphate (NADPH) as an electron donor. Changes in the intracellular glutathione/GSSG ratio or depletion of glutathione are usually associated with the occurrence or development of cell apoptosis (Baity et al., 2019). For example, the expression of glutathione reductase in the mitochondria increased significantly during carcinogenesis, but after TVN treatment, its expression decreased again (Figure 5A). The trend of differential metabolism between GSH and GSSG also confirms this (Supplementary Table S2). This indicates that during the carcinogenic process, cells increase the expression of GR in order to clear excess ROS. Due to the fact that TVN treatment can induce cancer cell apoptosis, resulting in a decrease in its number, the expression of GR decreases back to normal. The GO analysis of identified proteins of the A549 group *versus* the Beas-2B group is found in Supplementary Figure S7A.

### 3.7 Combined analysis of metabolome and proteome profiles

In order to associate the results of metabolome and proteome profiles, we mapped the differential proteins and metabolites to metabolic pathways. Then the first 10 KEGG pathways with the highest number of proteins and metabolites identified in this study were analyzed for visualization.

The results (Figure 6B) showed that these differential proteins and metabolites were mainly concentrated in the metabolic process such as purine metabolism, aminoacyl-tRNA biosynthesis, central carbon metabolism in cancer, arginine and proline metabolism, and PI3K-Akt signaling pathway. Further research found that most metabolites and proteins on the purine metabolism, arginine and proline metabolism, and PI3K-Akt signaling pathway of A549 cells were down-regulated after treatment with TVN, which implied that TVN could inhibit the normal metabolism of A549 cells and slow down the proliferation rate of A549 cells. The PI3K-Akt signaling pathway is widely deregulated in the human tumor spectrum (Liu et al., 2018). In addition, it promotes the cell cycle, cell proliferation, cell survival, and anti-apoptosis, which is achieved by activating Akt and downstream targets FOXO-1, GSK-3, etc. A study reported that ROS can promote the interaction between Akt-PP2A by affecting the binding activity of Akt-Hsp90, which in turn leads to a change in the conformation of Akt, and finally induces apoptosis by blocking Akt activation (Cao et al., 2009). Based on this, we have drawn an intact picture of the pathway that TVN may inhibit PI3K/Akt signaling pathway to increase apoptosis by inducing ROS accumulation in A549 cells. The combined analysis of metabolome and proteome profiles of A549 group verses Beas-2B group was found in Supplementary Figure S7B.

## 4 Conclusion

In summary, TVN could trigger ΔΨm reduction to improve intracellular ROS level increase, leading to apoptosis in A549 cells. Metabolomics and proteomics evaluations demonstrated that significantly identified differential proteins and metabolites are mainly related to metabolic pathways such as the PI3K/Akt signaling pathway. Therefore, we speculated that TVN could induce A549 cell apoptosis through ROS/PI3K/Akt pathway. These results should help to understand more about the apoptosis mechanism of A549 cells mediated by TVN and provide a strategy for the discovery of tumor therapeutic targets.

## Data Availability

The datasets presented in this study can be found in online repositories. The names of the repository/repositories and accession number(s) can be found in the article/Supplementary Material.

## References

[B1] Baity M. Wang L. Correa A.M. Zhang X. Zhang R. Pataer A. (2019). Glutathione reductase (GSR) gene deletion and chromosome 8 aneuploidy in primary lung cancers detected by fluorescence *in situ* hybridization. Am. J. Cancer Res. 9 (6), 1201–1211. 31285952 PMC6610060

[B2] Battistelli M. Malatesta M. Meschini S. (2016). Oxidative stress to promote cell death or survival. Oxid. Med. Cell Longev. 2016, 2054650–2054652. 10.1155/2016/2054650 26941887 PMC4753003

[B3] Beadsmoore C. J. Screaton N. J. (2003). Classification, staging and prognosis of lung cancer. Eur. J. Radio 45, 8–17. 10.1016/s0720-048x(02)00287-5 12499060

[B4] Bostanghadiri N. Pormohammad A. Chirani A. S. Pouriran R. Hashemi A. (2017). Comprehensive review on the antimicrobial potency of the plant polyphenol resveratrol. Biomed. Pharmacother. 95, 1588–1595. 10.1016/j.biopha.2017.09.084 28950659

[B5] Cao J. Xu D. Wang D. Wu R. Zhang L. Zhu H. (2009). ROS-driven Akt dephosphorylation at Ser-473 is involved in 4-HPR-mediated apoptosis in NB4 cells. Free Radic. Bio Med. 47, 536–547. 10.1016/j.freeradbiomed.2009.05.024 19482076

[B6] Chikara S. Nagaprashantha L. D. Singhal J. Horne D. Awasthi S. Singhal S. S. (2018). Oxidative stress and dietary phytochemicals:role in cancer chemoprevention and treatment. Cancer Lett. 413, 122–134. 10.1016/j.canlet.2017.11.002 29113871

[B7] Das D. K. Sato M. Ray P. S. Maulik G. Engelman R. M. Bertelli A. A. (1999). Cardioprotection of red wine: Role of polyphenolic antioxidants. Drugs Exp. Clin. Res. 25, 115–120. 10370873

[B8] Jiang Z. Chen K. Cheng L. Yan B. Qian W. Cao J. (2017). Resveratrol and cancer treatment: Updates. Ann. N. Y. Acad. Sci. 1403, 59–69. 10.1111/nyas.13466 28945938

[B9] Joo M.S. Kim W.D. Lee K.Y. Kim J.H. Koo J.H. Kim S.G. (2016). AMPK facilitates nuclear accumulation of nrf2 by phosphorylating at serine 550. Mol. Cell Biol. 36 (14), 1931–1942. 10.1128/MCB.00118-16 27161318 PMC4936058

[B10] Lang F. Qin Z. Li F. Zhang H. Hao E. (2015). Apoptotic cell death induced by resveratrol is partially mediated by the autophagy pathway in human ovarian cancer cells. PLoS ONE 10, e0129196. 10.1371/journal.pone.0129196 26067645 PMC4466135

[B11] Langcake P. Pryce R. J. (1977). Oxidative dimerisation of 4-hydroxystilbenes *in vitro*: Production of a grapevine phytoalexin mimic. Chem. Comm. 7, 208–210. 10.1039/c39770000208

[B12] Liu C. Xing J. Gao Y. (2018). UNBS5162 inhibits the proliferation of human A549 non-small-cell lung cancer cells by promoting apoptosis. Thorac. Cancer 9 (1), 105–111. 10.1111/1759-7714.12546 29130641 PMC5754305

[B13] Liu C.-Y. Lee C.-F. Wei Y.-H. (2009). Role of reactive oxygen species-elicited apoptosis in the pathophysiology of mitochondrial and neurodegenerative diseases associated with mitochondrial DNA mutations. J. Formos. Med. Assoc. 108, 599–611. 10.1016/s0929-6646(09)60380-6 19666347

[B14] Petrosillo G. Ruggiero F. M. Paradies G. (2004). Role of reactive oxygen species and cardiolipin in the release of cytochrome c from mitochondria. Faseb. J. 17, 2202–2208. 10.1096/fj.03-0012com 14656982

[B15] Poulsen M. M. Fjeldborg K. Ornstrup M. J. Kjar T. N. Nohr M. K. Pedersen S. B. (2015). Resveratrol and inflammation: Challenges in translating pre-clinical findings to improved patient outcomes. Biochim. Biophys. Acta 1852, 1124–1136. 10.1016/j.bbadis.2014.12.024 25583116

[B16] Samarghandian S. Azimi -Nezhad M. Farkhondeh T. Samini F. (2017). Anti-oxidative effects of curcumin on immobilization-induced oxidative stress in rat brain, liver and kidney. Biomed. Pharmacother. 87, 223–229. 10.1016/j.biopha.2016.12.105 28061405

[B17] Soden D.M. Dobson A.D. (2003). The use of amplified flanking region-PCR in the isolation of laccase promoter sequences from the edible fungus Pleurotus sajor-caju. J. Appl. Microbiol. 95 (3), 553–562. 10.1046/j.1365-2672.2003.02012.x 12911704

[B18] Vitrac X. Bornet A. Vanderlinde R. Valls J. Richard T. Delaunay J. C. (2005). Determination of stilbenes (Δ-Viniferin, trans-astringin, trans-piceid, cis-and trans-resveratrol, Ε-viniferin) in Brazilian wines. J. Agric. Food Chem. 53, 5664–5669. 10.1021/jf050122g 15998130

[B19] Wang L. Luo X. Pan Y. Zheng Z. Yin R. Tian X. (2021). Mechanism of laccase induction via emodin in *Trametes versicolor* . Front. Bioeng. Biotechnol. 9, 653800. 10.3389/fbioe.2021.653800 34095096 PMC8171328

[B20] Wang X.-A. Xiang S.-S. Li H.-F. Wu X.-S. Li M.-L. Shu Y.-J. (2014). Cordycepin induces S phase arrest and apoptosis in human gallbladder cancer cells. Molecules 19, 11350–11365. 10.3390/molecules190811350 25090123 PMC6271430

[B21] Wick G. Jansen-Durr P. Berger P. Blasko I. Grubeck-Loebenstein B. (2000). Diseases of aging. Vaccine 18, 1567–1583. 10.1016/s0264-410x(99)00489-2 10689131

[B22] Xue Y. Q. Di J. M. Luo Y. Cheng K. J. Wei X. Shi Z. (2014). Resveratrol oligomers for the prevention and treatment of cancers. Oxid. Med. Cell Longev. 2014, 765832. 10.1155/2014/765832 24799982 PMC3988857

[B23] Yin H. T. Tian Q. Z. Guan L. Zhou Y. Huang X. E. Zhang H. (2013). *In vitro* and *in vivo* evaluation of the antitumor efficiency of resveratrol against lung cancer. Asian pac. J. Cancer P 14, 1703–1706. 10.7314/apjcp.2013.14.3.1703 23679260

[B24] Zhan X.-R. Liao X.-Y. Luo X.-J. Zhu Y.-L. Feng S.-G. Yu C.-N. (2018). Comparative metabolomic and proteomic analyses reveal the regulation mechanism underlying MeJA-induced bioactive compound accumulation in cutleaf groundcherry (physalis angulata L) hairy roots. J. Agric.Food Chem. 66, 6336–6347. 10.1021/acs.jafc.8b02502 29874907

[B25] Zhang J.-Q. Li G.-P. Kang Y.-L. Teng B.-H. Yao C.-S. (2017). Biomimetic synthesis of resveratrol trimers catalyzed by horseradish peroxidase. Molecules 22, 819. 10.3390/molecules22050819 28513542 PMC6154677

[B26] Zhang L. Yang F. Cai J.-Y. Yang P. H. Liang Z. H. (2014). *In-situ* detection of resveratrol inhibition effect on epidermal growth factor receptor of living MCF-7 cells by Atomic Force Microscopy. Biosens. Bioelectron. 56, 271–277. 10.1016/j.bios.2014.01.024 24514079

[B27] Zhang Y.-Q. Yuan S. Pu J. Yang L.-N. Zhou X.-Y. Liu L.-X. (2017). Integrated metabolomics and proteomics analysis of Hippocampus in a rat model of depression. Neuroscience 371, 207–220. 10.1016/j.neuroscience.2017.12.001 29237567

[B28] Zheng Y. Cabassa-Hourton C. Planchais S. Lebreton S. Savouré A. (2021). The proline cycle as an eukaryotic redox valve. J. Exp. Bot. 72 (20), 6856–6866. 10.1093/jxb/erab361 34331757

[B29] Zhong Z. Li N. Liu L. He B. Igarashi Y. Luo F. (2018). Label-free differentially proteomic analysis of interspecific interaction between white-rot fungi highlights oxidative stress response and high metabolic activity. Fungal Biol. 122 (8), 774–784. 10.1016/j.funbio.2018.04.005 30007428

