# Supplementary figures and images for: The mechanism of trans-δ-viniferin inhibiting the proliferation of lung cancer cells A549 by targeting the mitochondria
Source: Front Pharmacol. 2023 May 18;14:1190127. doi: 10.3389/fphar.2023.1190127 (PMC10232840; doi:10.3389/fphar.2023.1190127)

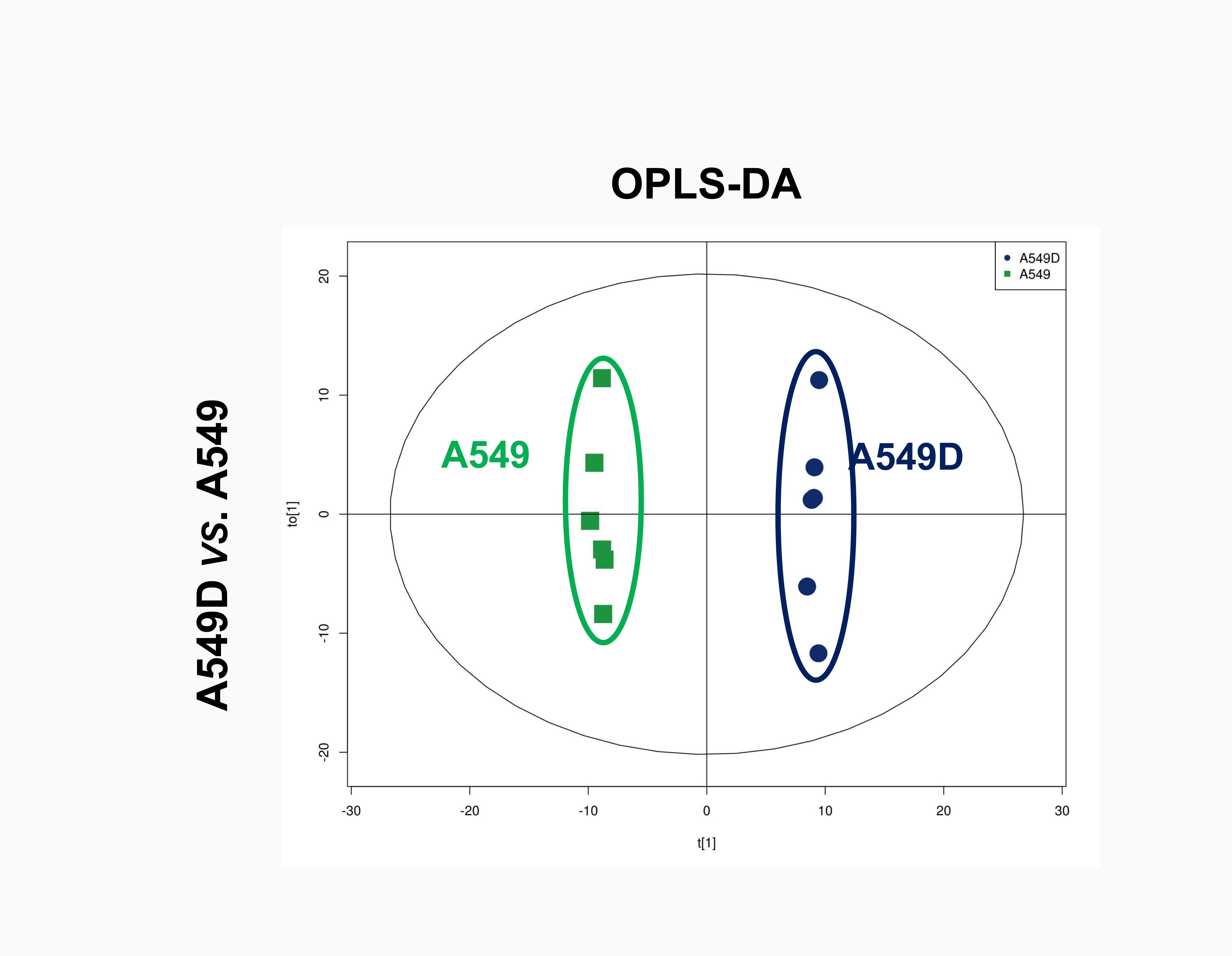

Supplement: Supplementary file 1 [file Image15.JPEG]

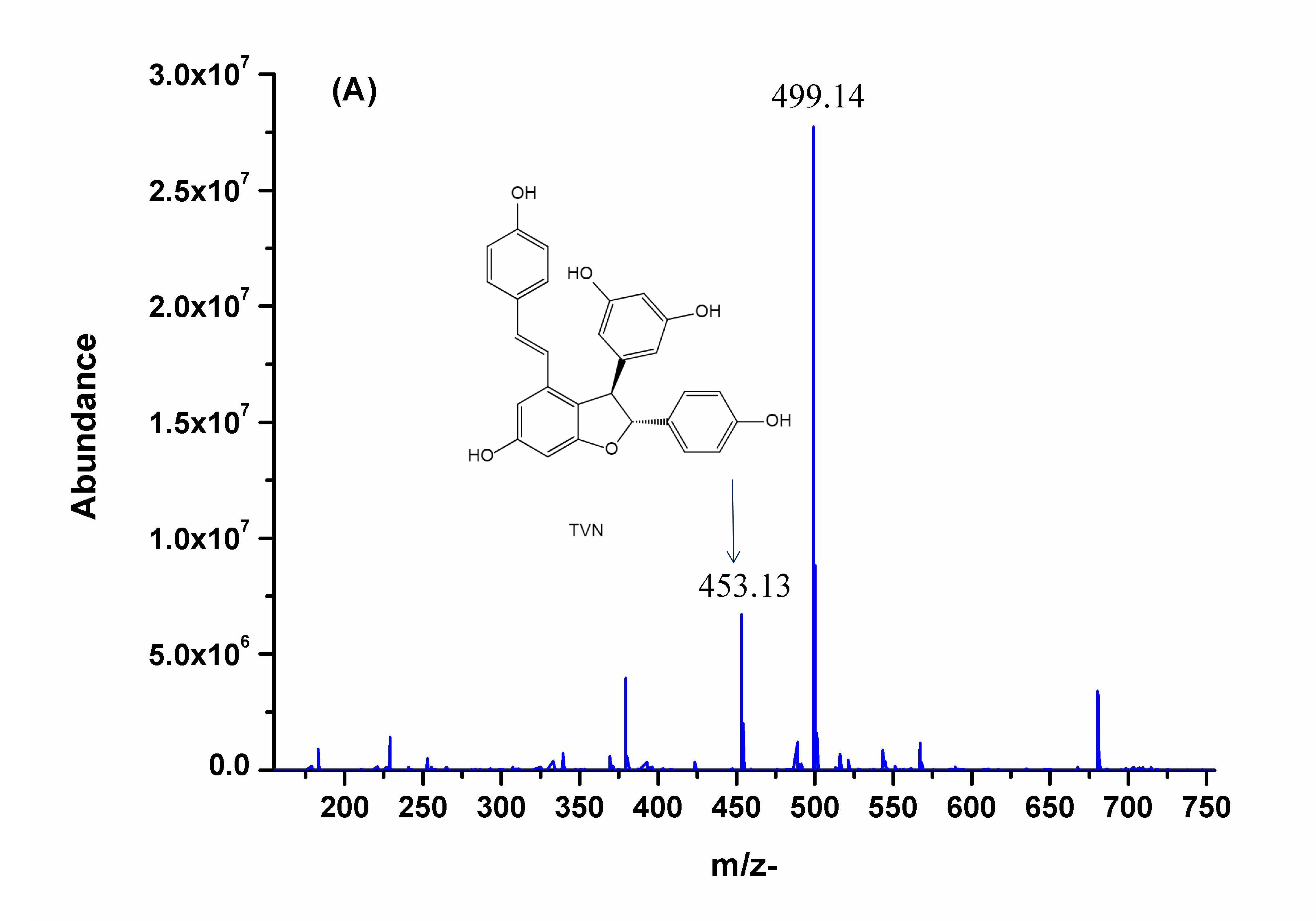

Supplement: Supplementary file 2 [file Image3.JPEG]

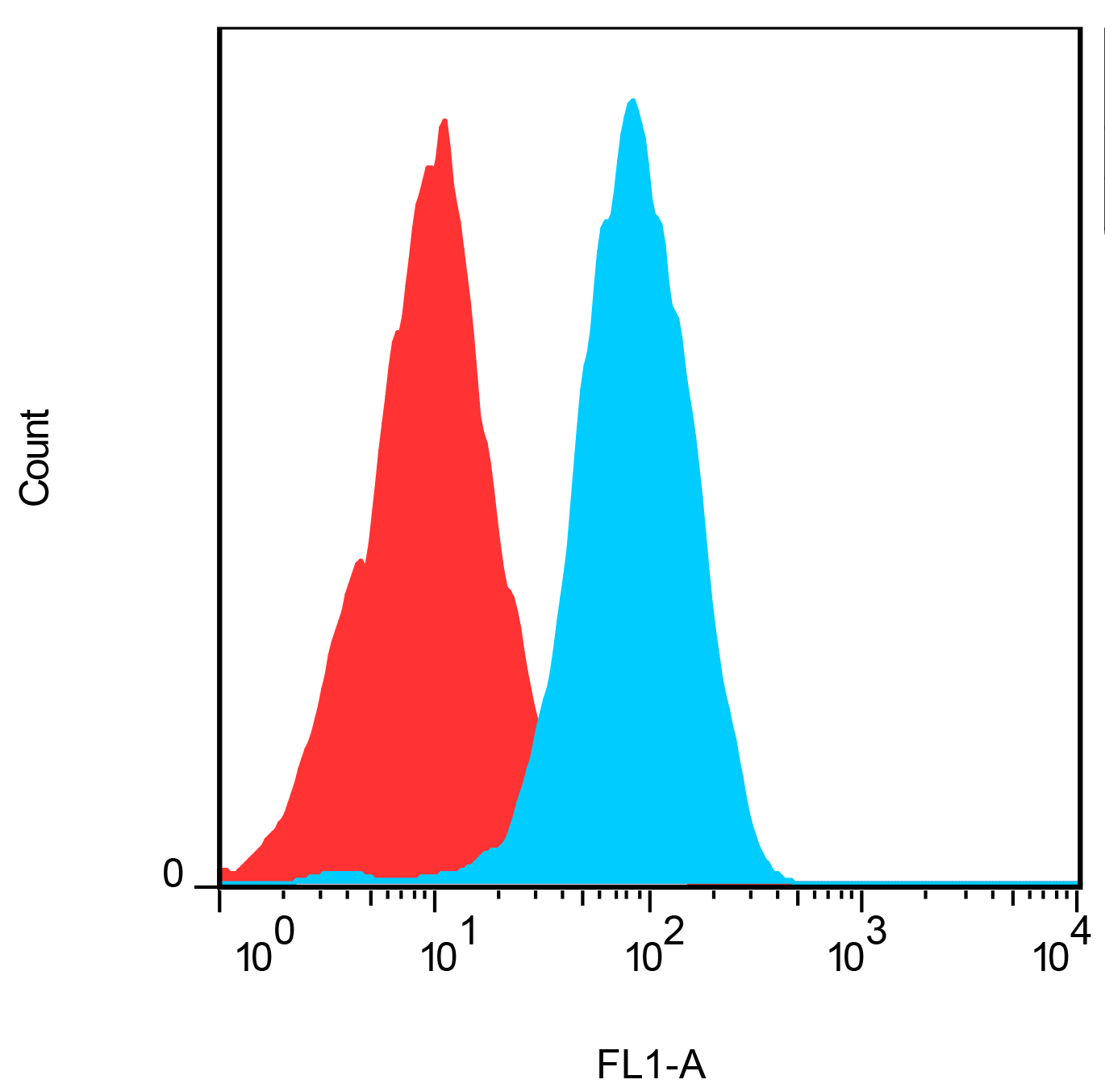

Supplement: Supplementary file 3 [file Image6.TIF]

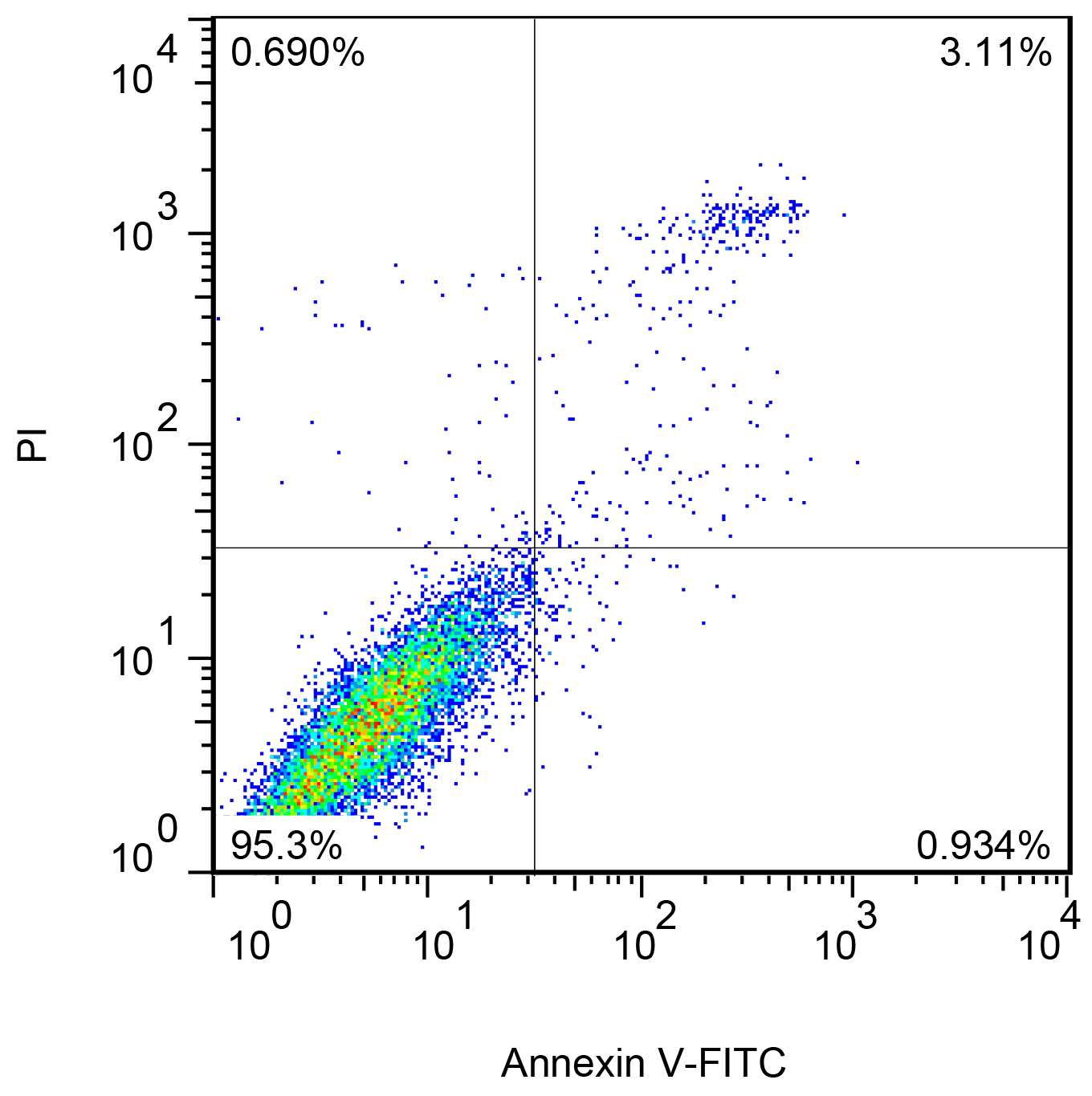

Supplement: Supplementary file 5 [file Image4.TIF]

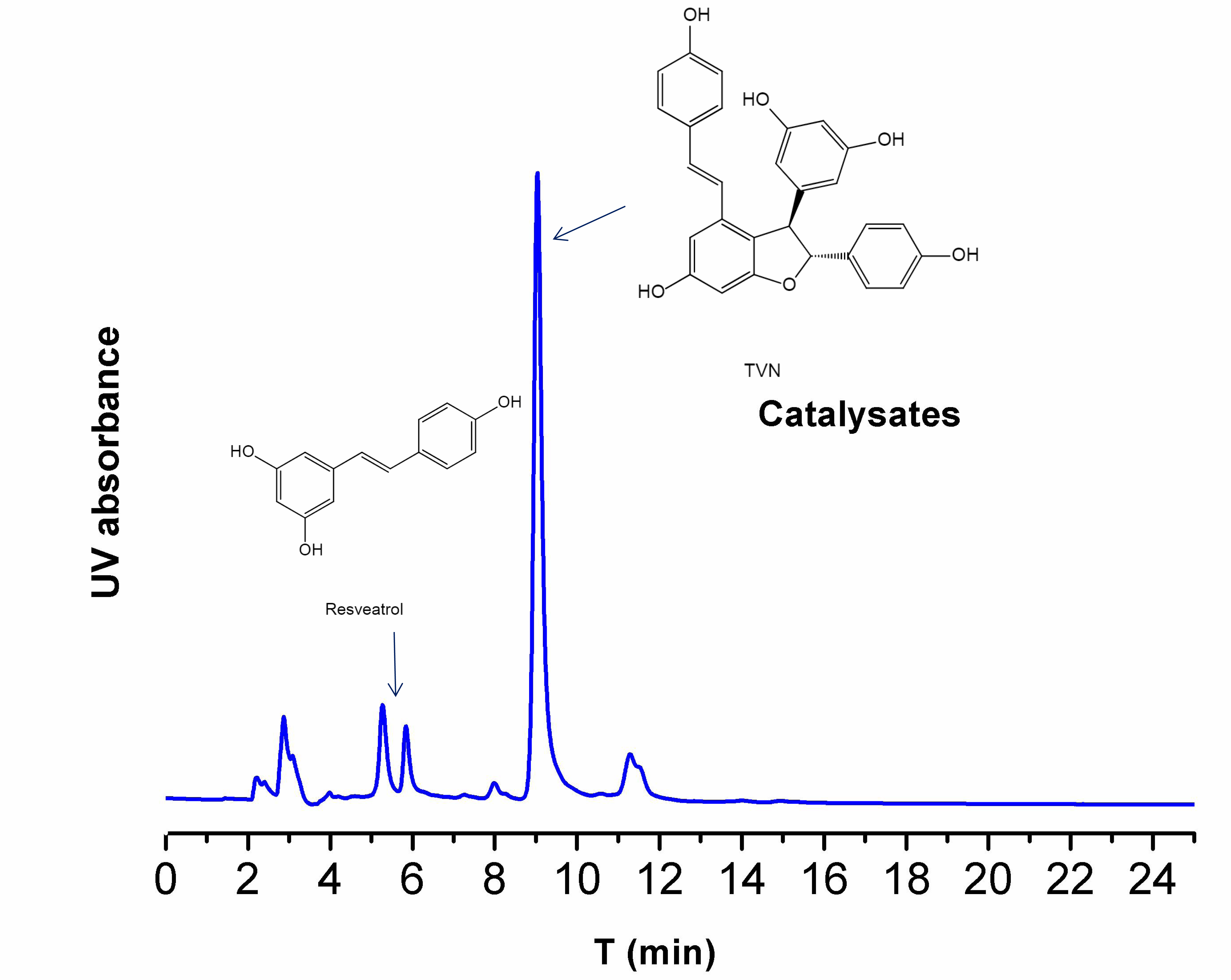

Supplement: Supplementary file 6 [file Image2.JPEG]

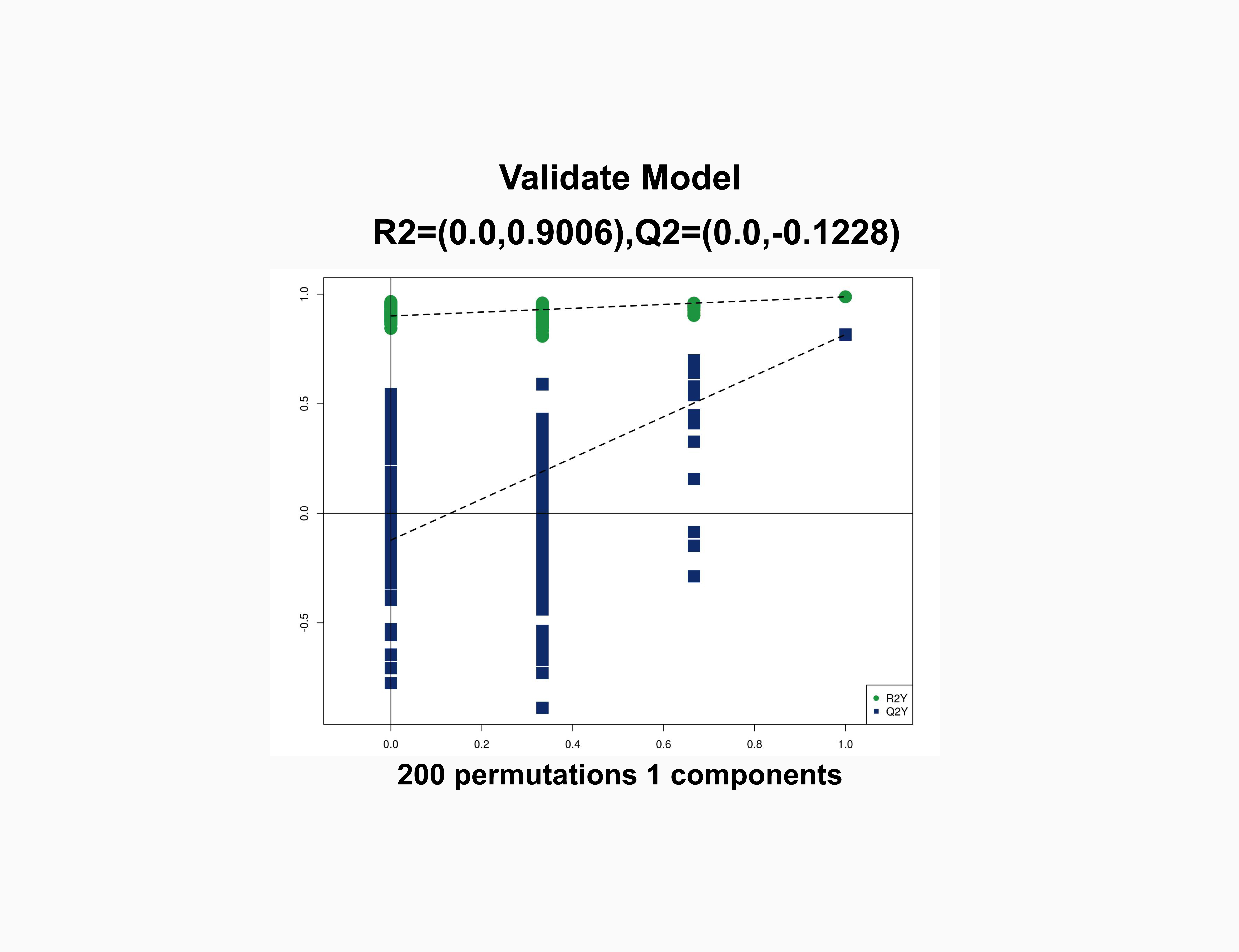

Supplement: Supplementary file 9 [file Image14.JPEG]

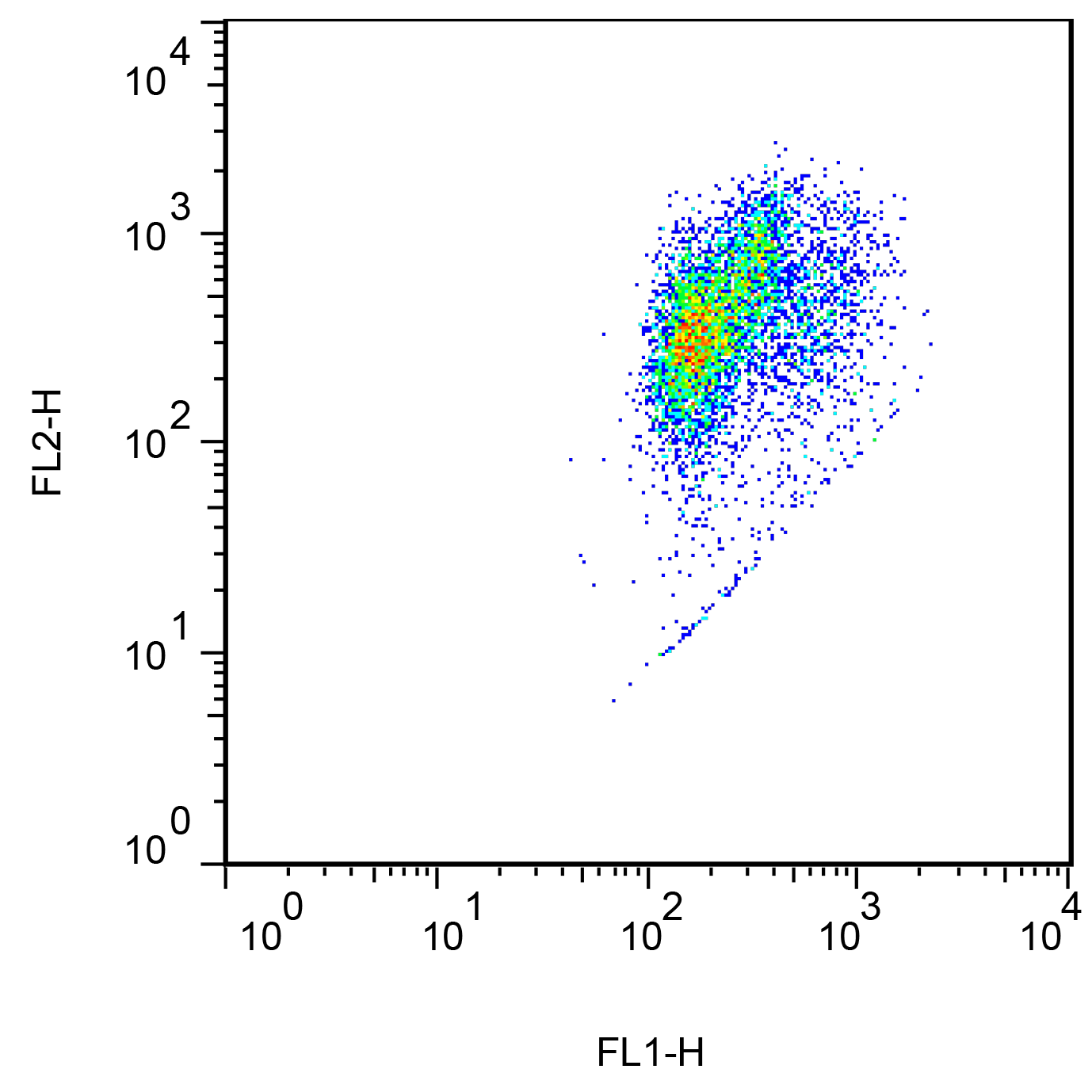

Supplement: Supplementary file 12 [file Image7.TIF]

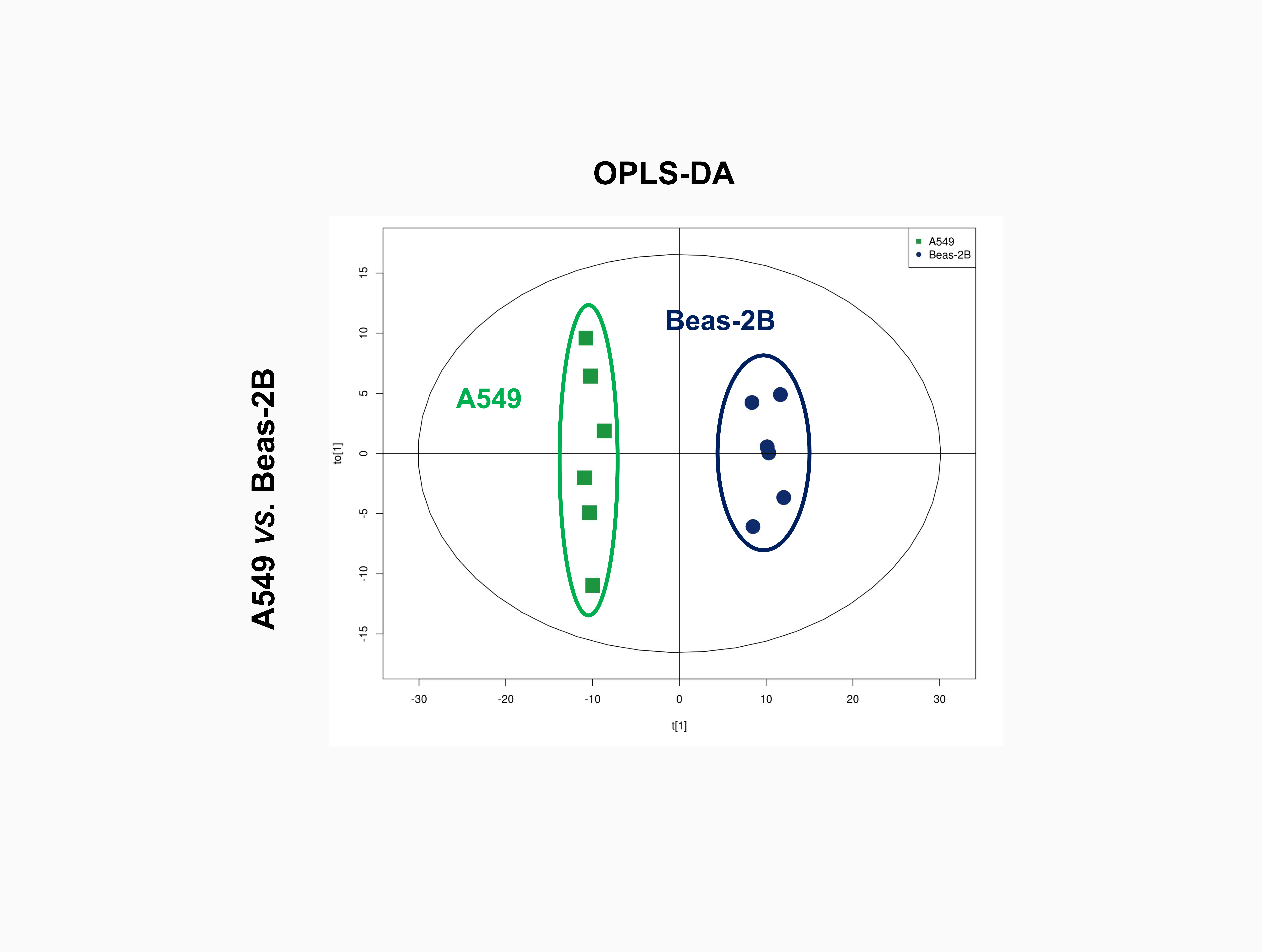

Supplement: Supplementary file 13 [file Image13.JPEG]

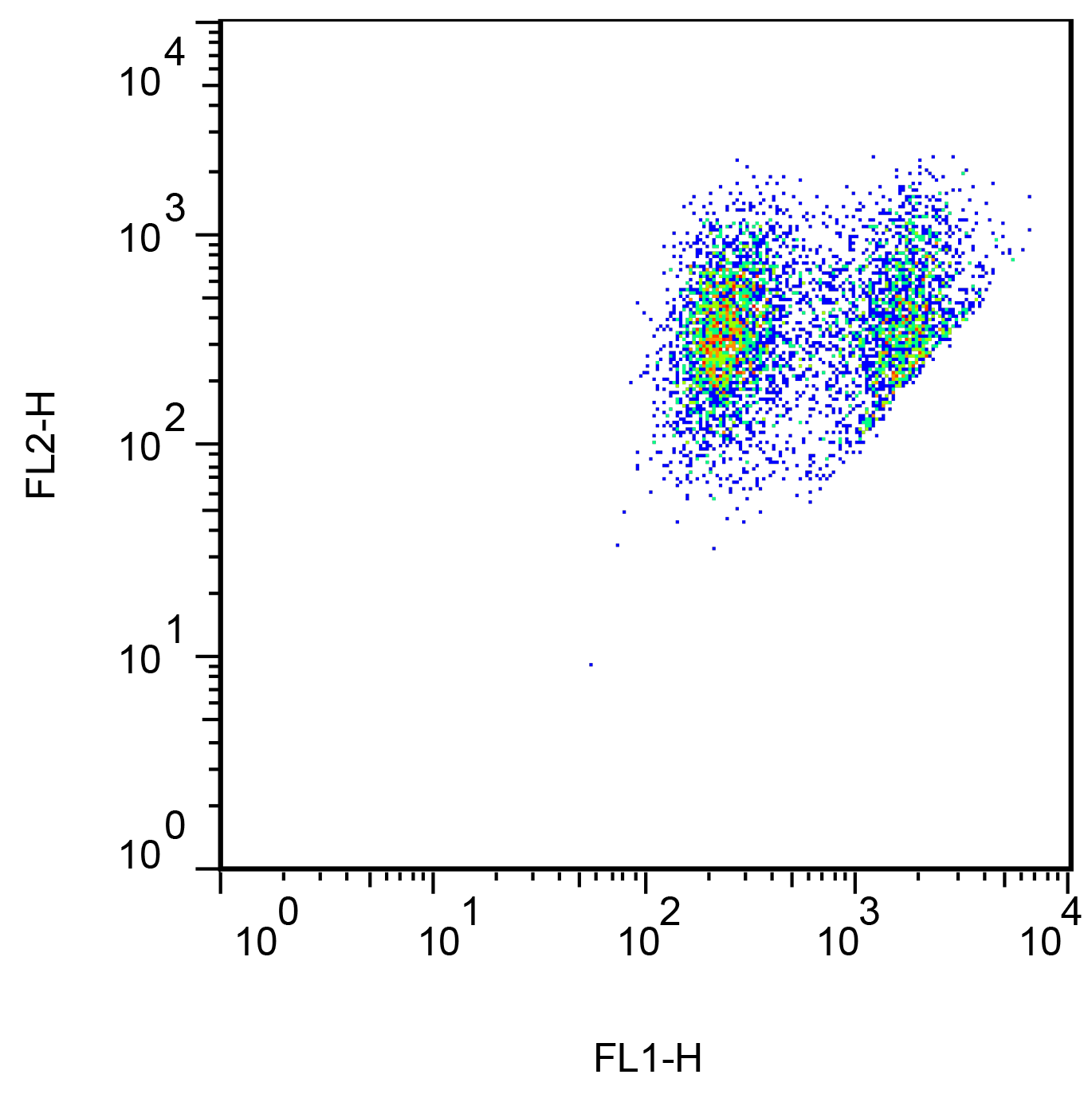

Supplement: Supplementary file 14 [file Image8.TIF]

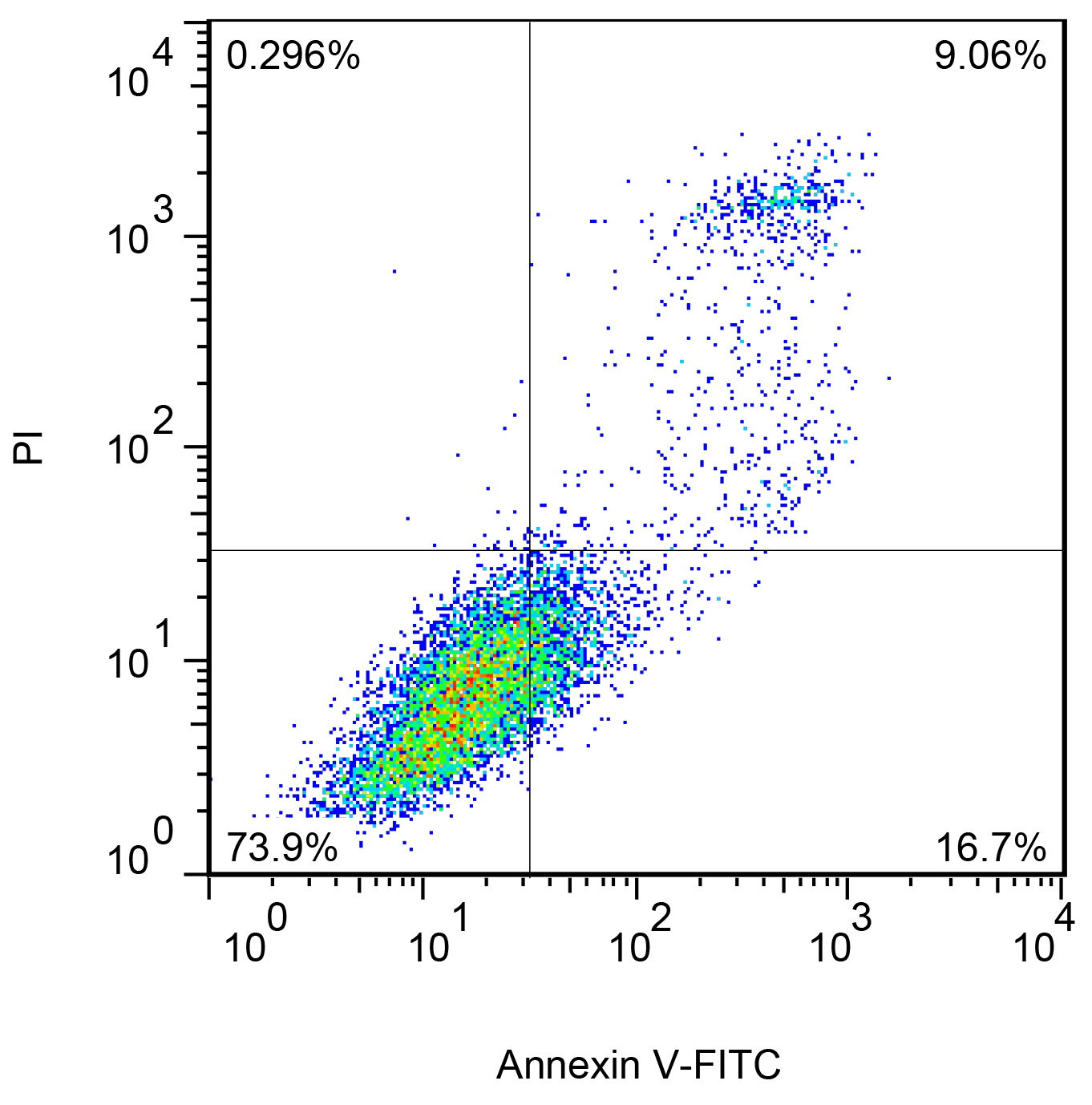

Supplement: Supplementary file 15 [file Image5.TIF]
