# Supplementary material for: The mechanism of trans-δ-viniferin inhibiting the proliferation of lung cancer cells A549 by targeting the mitochondria
Source: Front Pharmacol. 2023 May 18;14:1190127. doi: 10.3389/fphar.2023.1190127 (PMC10232840; doi:10.3389/fphar.2023.1190127)
Supplement: Supplementary file 16 [file DataSheet1.docx]

Supplementary Material

Mechanism of trans-δ-viniferin inhibiting proliferation of lung cancer cells A549 by targeting mitochondria

Ruochun Yin ^1,2^ ^†^, Yiling Zhang ^1^ ^†^, Liping Su ^1^, Dongdong Chen ^3^, Shidi Lou ^1^, Xuecai Luo ^2^, Lin Wang ^1,2^ ^*^, Rupei Tang^1^, Liang Zhang ^5^, Xiaohe Tian ^4^ ^*^

**† These authors contributed equally to this work.**

*** Correspondence:
Lin Wang:** [**wanglin@ahu.edu.cn**](mailto:wanglin@ahu.edu.cn)

**Xiaohe Tian:** [**xiaohe.t@wchscu.cn**](mailto:xiaohe.t@wchscu.cn)

# Materials and Methods

## Materials

Human A549 cell lines and human Beas-2B cell lines were both purchased from the cell bank of the Chinese Academy of Sciences (Shanghai, China). The cells were grown in RPMI-1640 (Hyclone Laboratories Inc., Logan, UT, USA) supplemented with 10% fetal bovine serum (Hyclone Laboratories Inc., Logan, UT, USA) and antibiotics (100 U/mL penicillin, 0.1 mg/mL streptomycin) (Hyclone Laboratories Inc., Logan, UT, USA). The medium was changed once every 48 hours, and human A549 cells were cultured in 37 ℃ 、5% CO2 incubator.

## Methods

### Untargeted metabolomics methods

#### Sample Collection and Preparation

We divided into three groups: Beas-2B group, A549 group and A549D group. Each group had seven parallel samples, and the number of cells in each sample was not less than 2*107. After they sticked to the wall,1640 serum-free medium containing 10 μM TVN was added and cultured for 60 h. Then the cells were washed twice with PBS and collected with cell scraping rods.

Every sample was slowly thawed at 4℃, mixed with 1 mL of cold methanol/acetonitrile/H2O (2:2:1, v/v/v) and adequately vortexed. The homogenate was sonicated at low temperature (30 min/once, twice), then incubated for 60 min at -20℃ to precipitate the protein, and centrifuged (13000 rpm, 4℃, 15 min). The supernatants were collected and dried under vacuum, and then stored at -80℃. The sample were redissolved in 100 μL acetonitrile/water (1:1, v/v), adequately vortexed again and then centrifuged (14000 rpm, 4℃, 15 min). The supernatants were collected for LC-MS/MS analysis.

### Label-free quantitative proteomics methods

#### Sample preparation for a label-free experiment

We divided into three groups: Beas-2B group, A549 group and A549D group. There were 7 parallel samples in each group, and the number of cells in each sample was not less than 2*107. After sticked to the wall, 1640 serum-free medium containing TVN was added and cultured for 60 h. Subsequently, the cells were washed twice with PBS and collected with cell scraping rods.

Cells were suspended on crushed ice in 200 μL lysis buffer (4% SDS, 100 mM DTT, 150 mM Tris-HCl，pH8.0) for cell lysis. Then cells were disrupted with agitation using a homogenizer (Fastprep-24®, MP Biomedical) and boiled for about 5 min. The samples were further ultrasonicated and boiled for another 5 min again. Undissolved cellular debris were carefully removed by centrifugation at 14 000 rpm for 15 min. The supernatant was collected and quantified with a BCA Protein Assay Kit (Bio-Rad, USA).

### Protein Digestion

Digestion of protein (250 μg for each sample) was performed according to the FASP procedure described by Wisniewski, Zougman et al. Briefly, the detergent, DTT and other low-molecular-weight components were carefully removed using 200 μL UA buffer (8 M Urea,150 mM Tris-HCl pH 8.0) by repeated ultrafiltration (Micro con units, 30 kD) facilitated by centrifugation. Then 100 μL 0.05 M iodoacetamide in UA buffer was added to block reduced cysteine residues and the samples were incubated for 20 min in darkness. The filter was washed with 100 μL UA buffer three times and then 100 μL 25 mM NH4HCO3 twice. Finally, the protein suspension was digested with 3 μg trypsin (Promega) in 40 μL 25 mM NH4HCO3 overnight at 37°C, and the resulting peptides were collected as a filtrate. The peptide content was estimated by UV light spectral density at 280 nm using an extinctions coefficient of 1.1 of 0.1% (g/L) solution that was calculated on the basis of the frequency of tryptophan and tyrosine in vertebrate proteins.

### Liquid Chromatography (LC) - Electrospray Ionization (ESI) Tandem MS (MS/MS)

**Analysis by Q Exactive**

The peptide of each sample was desalted on C18 Cartridges (Empore™ SPE Cartridges C18 (standard density), bed I.D. 7 mm, volume 3 ml, Sigma), then was concentrated by vacuum centrifugation and reconstituted in 40 µL of 0.1% (v/v) trifluoroacetic acid. MS experiments were performed on a Q Exactive mass spectrometer that was coupled to Easy nLC (Proxeon Biosystems, now Thermo Fisher Scientific). 5 μg peptide was loaded onto a the C18-reversed phase column (Thermo Scientific Easy Column, 10 cm long, 75 μm inner diameter, 3μm resin) in buffer A (2% acetonitrile and 0.1% Formic acid) and separated with a linear gradient of buffer B (80% acetonitrile and 0.1% Formic acid) at a flow rate of 250 nL/min controlled by Intelli Flow technology over 120 min. MS data was acquired using a data-dependent top10 method dynamically choosing the most abundant precursor ions from the survey scan (300–1800 m/z) for HCD fragmentation. Determination of the target value was based on predictive Automatic Gain Control (p AGC). Dynamic exclusion duration was 25 s. Survey scans were acquired at a resolution of 70000 at m/z 200 and resolution for HCD spectra was set to 17500 at m/z 200. Normalized collision energy was 30 eV and the underfill ratio, which specified the minimum percentage of the target value likely to be reached at maximum fill time, was defined as 0.1%. The instrument was run with peptide recognition mode enabled. Three mass spectrometry experiments were performed on each sample.

### Sequence Database Searching and Data Analysis

The MS data were analyzed using Max Quant software version 1.3.0.5. MS data were searched against the Human database (20244 total entries, downloaded 22/05/19). An initial search was set at a precursor mass window of 6 ppm. The search followed an enzymatic cleavage rule of Trypsin/P and allowed maximal two missed cleavage sites and a mass tolerance of 20 ppm for fragment ions. Carbamido methylation of cysteines was defined as fixed modification, while protein N-terminal acetylation and methionine oxidation were defined as variable modifications for database searching. The cutoff of global false discovery rate (FDR) for peptide and protein identification was set to 0.01.

Label-free quantification was carried out in Max Quant as previously described. Protein aboundance was calculated on the basis of the normalized spectral protein intensity (LFQ intensity).

### Data processing

The raw MS data (wiff. scan files) were converted to MzXML files using Proteo Wizard MS Convert before importing into freely available XCMS software. The following parameters were used to pick peak: cent Wave m/z = 25 ppm, peak width = c (10, 60), prefilter = c (10, 100). For peak grouping, bw = 5, mzwid = 0.025, minfrac = 0.5 were used. CAMERA (Collection of Algorithms of metabolite profile Annotation) was sued for annotation of isotopes and adducts. In the extracted ion features, only the variables having more than 50% of the nonzero measurement values in at least one group were retained. The compound identification of metabolites was performed by comparing of accuracy m/z value (< 25 ppm) and the MS / MS spectrum with an internal database established according to existing real standards.

### Statistical analysis

After normalizing the processed data to the total peak intensity, it was uploaded to Metabo Analyst software for further analysis. Principal component analysis (PCA) and partial least square discriminant analysis (OPLS-DA) were performed for positive and negative models after log transformation and Pareto-scaling. The variable importance in the projection (VIP) value of each variable in the OPLS-DA model was calculated to indicate its contribution to the classification. Metabolites with the VIP value >1 was further applied to Student’s t-test at univariate level to measure the significance of each metabolite and the p values less than 0.05 were considered as statistically significant.

# Supplementary Figures and Tables

**Table S1.** Differential metabolites in positive and negative ion modes between A549D and A549 groups.

|  | **POS** | | | |
| --- | --- | --- | --- | --- |
| **NO.** | **description** | **VIP** | **FC** | **p-value** |
| **1** | Lumichrome | 1.11054871 | 0.19219518 | 1.7053E-08 |
| **2** | N-Acetyl-D-glucosamine | 1.23240288 | 1.86301457 | 4.0092E-05 |
| **3** | Phosphocreatine | 1.82754521 | 0.29232104 | 4.4996E-05 |
| **4** | 3-Methyluridine | 1.34318979 | 0.05821051 | 5.1778E-05 |
| **5** | 2-Methylbutyroylcarnitine | 7.82506105 | 0.27455121 | 5.9084E-05 |
| **6** | 2-Hydroxyadenine | 1.88175192 | 0.31753182 | 6.484E-05 |
| **7** | Thiamine monophosphate | 1.17354619 | 0.31267027 | 0.00013982 |
| **8** | Guanosine | 2.28064819 | 0.31181616 | 0.0001806 |
| **9** | Cytosine | 3.20864942 | 0.24850136 | 0.00043477 |
| **10** | Cytidine | 3.23696581 | 0.24016884 | 0.0004704 |
| **11** | Glutathione disulfide | 7.73616639 | 2.69603727 | 0.00050475 |
| **12** | N6-methyladenosine | 4.70380722 | 0.2543443 | 0.00054483 |
| **13** | Thiamine pyrophosphate (TPP) | 1.43320984 | 0.33952203 | 0.00066901 |
| **14** | D-Biotin | 1.02925907 | 0.29750942 | 0.00068082 |
| **15** | Phosphorylcholine | 14.6025318 | 0.48434664 | 0.00068765 |
| **16** | UDP-N-acetylglucosamine | 1.80855843 | 1.67493332 | 0.00129146 |
| **17** | (-)-Riboflavin | 1.79709457 | 0.31715372 | 0.00140511 |
| **18** | Adenosine monophosphate (AMP) | 1.59696808 | 2.28931622 | 0.00339085 |
| **19** | L-Cystine | 1.85707339 | 0.11261917 | 0.00428962 |
| **20** | Guanosine 5'-monophosphate (GMP) | 2.02886352 | 2.40353357 | 0.00432556 |
| **21** | Taurine | 4.62016224 | 0.68822615 | 0.00469201 |
| **22** | Glutathione | 4.4269272 | 36.9858011 | 0.00471263 |
| **23** | 1-Methylnicotinamide | 4.55466138 | 9.84900906 | 0.00627387 |
| **24** | N6-Methyladenine | 1.05067425 | 0.27735028 | 0.00716646 |
| **25** | L-Glutamate | 4.38376033 | 0.61591915 | 0.00768181 |
| **26** | Adenosine 5'-phosphosulfate (APS) | 5.55660619 | 2.48556692 | 0.00856422 |
| **27** | Adenosine | 1.88812962 | 0.33505174 | 0.00892756 |
| **28** | 2-Phenylacetamide | 1.80293264 | 0.42245286 | 0.00916969 |
| **29** | Tyr-Ser | 4.36869768 | 0.36553928 | 0.00976949 |
| **30** | Deoxyadenosine | 8.84621835 | 0.11082481 | 0.01102744 |
| **31** | 1-Aminocyclopropanecarboxylic acid | 1.03355963 | 0.64189399 | 0.01279694 |
| **32** | Ribothymidine | 1.33439826 | 0.19625778 | 0.01284699 |
| **33** | 2'-O-methyladenosine | 2.62475264 | 0.47748213 | 0.01533398 |
| **34** | Acetylcarnitine | 2.43141274 | 0.50328294 | 0.01534276 |
| **35** | L-Pyroglutamic acid | 2.62038549 | 0.66207276 | 0.01561244 |
| **36** | Nicotinamide | 9.2170862 | 1.26127255 | 0.01752501 |
| **37** | 1-Deoxynojirimycin | 1.23141088 | 0.54112351 | 0.01824974 |
| **38** | Riboflavin | 1.16323149 | 0.37365317 | 0.01849847 |
| **39** | Nicotinate | 1.69524108 | 0.55948161 | 0.01952625 |
| **40** | Thioetheramide-PC | 2.79353902 | 0.45686702 | 0.02093417 |
| **41** | Nicotinate D-ribonucleotide | 1.36334677 | 0.48265758 | 0.02165341 |
| **42** | 1,2-dioleoyl-sn-glycero-3-phosphatidylcholine | 1.82719854 | 0.4004348 | 0.02861584 |
| **43** | Uridine 5'-diphosphate (UDP) | 1.40554633 | 2.17099447 | 0.0338119 |
| **44** | L-Palmitoylcarnitine | 1.20025301 | 0.2877394 | 0.03437322 |
| **45** | Guanosine 5'-diphosphate (GDP) | 1.3086472 | 2.7813797 | 0.03884072 |
| **46** | L-Phenylalanine | 1.10731427 | 0.56912588 | 0.04044748 |
| **47** | Uracil | 1.31794718 | 0.20041229 | 0.04196312 |
| **48** | beta-Nicotinamide D-ribonucleotide | 1.86619994 | 0.30180325 | 0.04531504 |
| **49** | Thymine | 3.16668098 | 0.06552838 | 0.04931478 |
|  | **NEG** | | | |
| **NO.** | **description** | **VIP** | **FC** | **p-value** |
| **1** | Valeric acid | 1.4040699 | 0.42126512 | 7.1333E-07 |
| **2** | 3-Methyluridine | 2.6029335 | 0.02792996 | 1.7947E-06 |
| **3** | Myristoleic acid | 1.5886057 | 0.3690069 | 2.2707E-06 |
| **4** | Lumichrome | 1.8737281 | 0.33303695 | 2.6935E-06 |
| **5** | Uridine diphosphate-N-acetylglucosamine | 4.48731 | 2.04075144 | 1.1327E-05 |
| **6** | 2'-O-Methyluridine | 1.3672905 | 0.29050684 | 5.7769E-05 |
| **7** | Cytidine | 2.5083279 | 0.14615667 | 5.9007E-05 |
| **8** | 4-thiouridine | 1.6840341 | 0.19476957 | 6.8599E-05 |
| **9** | DL-lactate | 5.8502091 | 6.26481165 | 7.3137E-05 |
| **10** | Thymine | 2.1241712 | 0.09048039 | 0.00014792 |
| **11** | Guanosine | 4.8358844 | 0.30586233 | 0.00025463 |
| **12** | ketoisocaproic acid | 6.870843 | 3.3463495 | 0.00042815 |
| **13** | L-Glutamine | 1.2693682 | 3.74354345 | 0.00047414 |
| **14** | myo-Inositol | 2.9121404 | 0.59146191 | 0.00048772 |
| **15** | L-Cystine | 1.4482612 | 0.09247583 | 0.00056786 |
| **16** | Pantetheine | 1.1994521 | 13.6940487 | 0.00070946 |
| **17** | Glutathione disulfide | 8.5306954 | 3.21315991 | 0.00094732 |
| **18** | Phthalic acid Mono-2-ethylhexyl Ester | 1.192604 | 0.53536713 | 0.00125111 |
| **19** | Eicosapentaenoic acid | 2.0263436 | 0.36416652 | 0.00126053 |
| **20** | Guanosine 5'-monophosphate (GMP) | 1.7439217 | 2.6470324 | 0.00137143 |
| **21** | Pantothenate | 1.8378166 | 5.08703157 | 0.00183694 |
| **22** | L-Glutamate | 7.452822 | 0.55958008 | 0.00188206 |
| **23** | Uridine | 1.2903804 | 0.28291108 | 0.00206381 |
| **24** | Taurine | 4.8154973 | 0.64862932 | 0.00207191 |
| **25** | Adenosine | 3.0694948 | 0.39412358 | 0.00215994 |
| **26** | Guanosine 5'-diphosphate (GDP) | 1.3032811 | 3.22158316 | 0.00216441 |
| **27** | Prostaglandin E2 | 2.0792025 | 0.03707055 | 0.00224203 |
| **28** | D-Sorbitol | 2.8885425 | 1.6323489 | 0.00229863 |
| **29** | N-Acetylglucosamine 1-phosphate | 1.5316018 | 0.47044825 | 0.00266978 |
| **30** | Adenosine monophosphate (AMP) | 4.7846437 | 2.84605974 | 0.002815 |
| **31** | cis-9-Palmitoleic acid | 2.8983379 | 0.4919683 | 0.00309107 |
| **32** | 8-iso-Prostaglandin A2 | 1.0166737 | 0.08805919 | 0.00522188 |
| **33** | Adenosine 5'-diphosphate (ADP) | 1.205172 | 2.93667542 | 0.00565854 |
| **34** | Uracil | 1.4611988 | 0.27423832 | 0.00633706 |
| **35** | Orotate | 1.346712 | 0.28127984 | 0.00676557 |
| **36** | 1-Palmitoyl-2-oleoyl-phosphatidylglycerol | 2.9593323 | 0.29766139 | 0.00699631 |
| **37** | 1-Methylpseudouridine | 1.2051541 | 0.21557676 | 0.00779405 |
| **38** | Deoxyguanosine triphosphate (dGTP) | 2.3035295 | 2.69675924 | 0.01256737 |
| **39** | Dodecanoic acid | 1.5490661 | 0.71443732 | 0.01333152 |
| **40** | Pyridoxal (Vitamin B6) | 2.4183666 | 0.39870625 | 0.01499111 |
| **41** | Glutathione | 1.0900343 | 6.52909593 | 0.01546985 |
| **42** | Uridine diphosphate glucose (UDP-D-Glucose) | 1.9384163 | 2.16713173 | 0.017541 |
| **43** | Alpha-D-Glucose | 1.22906 | 3.99773922 | 0.01807064 |
| **44** | L-Phenylalanine | 1.3575197 | 0.65116137 | 0.01995673 |
| **45** | S-Methyl-5'-thioadenosine | 1.8529649 | 1.41895243 | 0.03231883 |
| **46** | Cytidine 5'-diphosphate (CDP) | 1.2270232 | 1.78322933 | 0.03365716 |
| **47** | Phosphoenolpyruvate | 1.3042411 | 0.60886233 | 0.03508712 |
| **48** | D-Proline | 2.3280276 | 0.54867403 | 0.04495544 |
| **49** | L-Threonate | 2.3426794 | 0.39481431 | 0.04624823 |
| **50** | Dihydroxyacetone phosphate | 1.0493036 | 0.41711469 | 0.04821933 |

VIP, variable importance in the projection; FC, fold change.

FC was calculated as the ratio of the average variable value in the A549D group to that in the A549 group.

1. **Table S2.** Differential metabolites in positive and negative ion modes between A549 and Beas-2B groups.

|  | **POS** | | | | | | |  |
| --- | --- | --- | --- | --- | --- | --- | --- | --- |
| **NO.** | **description** | **VIP** | | **FC** | | **p-value** | |  |
| **1** | Lys-Leu | 1.474074 | | 0.06352718 | | 8.743E-12 | |  |
| **2** | beta-Nicotinamide D-ribonucleotide | 9.509109 | | 0.04404614 | | 7.4896E-07 | |  |
| **3** | L-Citrulline | 1.361175 | | 22.4256233 | | 2.1098E-06 | |  |
| **4** | Creatine | 1.645661 | | 0.07287848 | | 4.5755E-06 | |  |
| **5** | 1-Deoxynojirimycin | 1.611989 | | 76.1535331 | | 7.1066E-06 | |  |
| **6** | N-Acetyl-D-glucosamine | 2.084766 | | 0.17363092 | | 7.7958E-06 | |  |
| **7** | Cytosine | 2.972866 | | 85.3954095 | | 7.8965E-06 | |  |
| **8** | UDP-N-acetylglucosamine | 2.86255 | | 0.2483354 | | 8.1246E-06 | |  |
| **9** | Cytidine | 2.982039 | | 133.849326 | | 9.3824E-06 | |  |
| **10** | 2'-Deoxyinosine | 1.084438 | | 0.21694048 | | 1.8931E-05 | |  |
| **11** | Maltotriose | 1.261516 | | 0.10418457 | | 2.6449E-05 | |  |
| **12** | Cytidine monophosphate N-acetylneuraminic acid | 1.870491 | | 0.13026187 | | 2.8949E-05 | |  |
| **13** | Nicotinamide | 4.092632 | | 0.03557556 | | 4.1011E-05 | |  |
| **14** | L-Pipecolic acid | 1.225594 | | 0.330996 | | 4.4122E-05 | |  |
| **15** | Nicotinate D-ribonucleotide | 9.13888 | | 0.03202229 | | 6.1886E-05 | |  |
| **16** | L-Cysteine | 1.119687 | | 7.0255855 | | 6.6434E-05 | |  |
| **17** | Creatinine | 5.119782 | | 0.05603842 | | 0.00010282 | |  |
| **18** | Glutathione | 2.130471 | | 0.07087015 | | 0.00016787 | |  |
| **19** | 2-Methylbutyroylcarnitine | 5.538171 | | 2.40504884 | | 0.00032665 | |  |
| **20** | Phosphocreatine | 4.732468 | | 0.1093818 | | 0.00042005 | |  |
| **21** | Uracil | 2.874284 | | 0.13817342 | | 0.00046651 | |  |
| **22** | gamma-L-Glutamyl-L-glutamic acid | 5.138182 | | 3.29472472 | | 0.00050996 | |  |
| **23** | Adenosine 3',5'-diphosphate (PAP) | 1.853187 | | 0.02028983 | | 0.00064654 | |  |
| **24** | 1-Stearoyl-2-oleoyl-sn-glycerol 3-phosphocholine (SOPC) | 1.062232 | | 4.12597412 | | 0.0017159 | |  |
| **25** | 3-Phospho-D-glycerate | 1.679559 | | 0.16088007 | | 0.00198376 | |  |
| **26** | L-Cystine | 1.645366 | | 23.8529553 | | 0.00225367 | |  |
| **27** | L-Tyrosine | 1.461275 | | 0.50741769 | | 0.00230809 | |  |
| **28** | Allopurinol riboside | 2.029481 | | 0.19282486 | | 0.00283728 | |  |
| **29** | Thymidine | 1.404259 | | 0.18705531 | | 0.00309654 | |  |
| **30** | 2'-O-methylguanosine | 1.712477 | | 0.19741821 | | 0.00397108 | |  |
| **31** | N-Acetyl-D-Glucosamine 6-Phosphate | 1.05293 | | 0.07757423 | | 0.00417773 | |  |
| **32** | Thioetheramide-PC | 2.758832 | | 3.41485858 | | 0.00425932 | |  |
| **33** | Hypoxanthine | 11.81132 | | 0.32011171 | | 0.00485309 | |  |
| **34** | Glycerophosphocholine | 3.881185 | | 1.76679969 | | 0.00599478 | |  |
| **35** | S-Methyl-5'-thioadenosine | 5.04103 | | 0.67155439 | | 0.00612364 | |  |
| **36** | Nicotinic acid adenine dinucleotide (NAAD) | 1.87561 | | 0.12875453 | | 0.00619502 | |  |
| **37** | Glutathione disulfide | 3.445714 | | 0.56224264 | | 0.00857813 | |  |
| **38** | Inosine | 3.645455 | | 0.4209903 | | 0.00899364 | |  |
| **39** | Nicotinate | 1.634398 | | 2.71650854 | | 0.01012104 | |  |
| **40** | S-Adenosylmethionine | 1.604472 | | 0.51525269 | | 0.01141133 | |  |
| **41** | 2'-O-methylinosine | 1.931234 | | 0.09719549 | | 0.01460444 | |  |
| **42** | L-Pyroglutamic acid | 2.323091 | | 1.41977223 | | 0.01616589 | |  |
| **43** | Taurine | 3.199243 | | 1.14358204 | | 0.01834454 | |  |
| **44** | 1-Aminocyclopropanecarboxylic acid | 1.165993 | | 1.38750385 | | 0.0187151 | |  |
| **45** | L-Isoleucine | 1.559727 | | 0.46450744 | | 0.02060807 | |  |
| **46** | 2-Hydroxyadenine | 1.411886 | | 0.12071831 | | 0.0228761 | |  |
| **47** | L-Glutamate | 3.588929 | | 1.41797211 | | 0.02343698 | |  |
| **48** | Ribothymidine | 1.600607 | | 0.29793318 | | 0.02383591 | |  |
| **49** | Choline | 3.320126 | | 0.75865375 | | 0.02494698 | |  |
| **50** | Uridine | 1.073244 | | 0.64237607 | | 0.02765037 | |  |
| **51** | 1-Methyladenosine | 1.034222 | | 0.31674693 | | 0.02775414 | |  |
| **52** | 1-Oleoyl-sn-glycero-3-phosphocholine | 1.957695 | | 2.18893934 | | 0.02924342 | |  |
| **53** | Thymine | 3.201495 | | 0.33921397 | | 0.02978851 | |  |
| **54** | Guanosine 5'-monophosphate (GMP) | 1.11337 | | 3.25573512 | | 0.03175955 | |  |
| **55** | Xanthine | 2.103724 | | 0.09131905 | | 0.03373195 | |  |
| **56** | Glycerol 3-phosphate | 1.041266 | | 0.21173173 | | 0.03561666 | |  |
| **57** | Adenosine | 2.451317 | | 2.57710687 | | 0.03614232 | |  |
| **58** | Nicotinamide adenine dinucleotide (NAD) | 2.103929 | | 0.22811953 | | 0.03647492 | |  |
| **59** | L-Phenylalanine | 1.889941 | | 0.50841561 | | 0.03916687 | |  |
| **60** | Adenine | 1.98449 | | 0.41074029 | | 0.04306023 | |  |
| **61** | Guanosine | 1.842386 | | 0.28947235 | | 0.0474212 | |  |
|  | **NEG** | | | | | | | |
| **NO** | **description** | | **VIP** | | **FC** | | **p-value** | |
| **1** | L-Citrulline | | 2.319403 | | 41.6158004 | | 8.7071E-09 | |
| **2** | Nicotinate D-ribonucleotide | | 2.199762 | | 0.0164844 | | 1.0068E-08 | |
| **3** | L-Rhamnose | | 2.985055 | | 10.8743032 | | 3.1315E-08 | |
| **4** | D-Quinovose | | 2.487368 | | 10.4865258 | | 1.2965E-06 | |
| **5** | L-Fucose-1-phosphate | | 3.401108 | | 17.4088854 | | 2.5877E-06 | |
| **6** | N-Acetylneuraminic acid | | 1.18969 | | 0.0642938 | | 2.8509E-06 | |
| **7** | Uridine diphosphate-N-acetylglucosamine | | 14.67044 | | 0.05357522 | | 4.9968E-06 | |
| **8** | L-Arginine | | 2.582593 | | 0.05041725 | | 1.0296E-05 | |
| **9** | N-Acetyl-L-alanine | | 1.094278 | | 3.99251508 | | 1.0482E-05 | |
| **10** | myo-Inositol | | 4.393924 | | 0.4045955 | | 1.286E-05 | |
| **11** | Cytidine | | 2.416102 | | 17.9788616 | | 1.3635E-05 | |
| **12** | Thymine | | 1.76324 | | 0.21306484 | | 2.1467E-05 | |
| **13** | D-Fructose | | 1.362554 | | 0.04538311 | | 4.2998E-05 | |
| **14** | Deoxyinosine | | 7.424778 | | 0.2129131 | | 0.00013714 | |
| **15** | D-Sorbitol | | 3.088844 | | 9.42706779 | | 0.00014515 | |
| **16** | Myristoleic acid | | 1.0352 | | 0.53463513 | | 0.00029665 | |
| **17** | L-Cystine | | 1.336343 | | 15.3020237 | | 0.0003987 | |
| **18** | Phosphocreatine | | 1.904846 | | 0.10613048 | | 0.00048411 | |
| **19** | Cholesterol 3-sulfate | | 2.247948 | | 24.7255247 | | 0.00050467 | |
| **20** | D-Ribulose 5-phosphate | | 2.289442 | | 0.04208679 | | 0.00052417 | |
| **21** | 3-Methyluridine | | 1.73758 | | 2.15020677 | | 0.00059562 | |
| **22** | Gossypol | | 1.917969 | | 0.1259878 | | 0.00064534 | |
| **23** | Xanthine | | 2.371281 | | 0.13163972 | | 0.0006831 | |
| **24** | Uridine 5'-diphosphoglucuronic acid (UDP-D-glucuronate) | | 5.521092 | | 2.02611534 | | 0.00069324 | |
| **25** | N-Acetylaspartylglutamate (NAAG) | | 1.371456 | | 0.56752442 | | 0.00100963 | |
| **26** | Cytidine 5'-monophosphate (CMP) | | 1.342533 | | 0.07181841 | | 0.00104474 | |
| **27** | Adenine | | 1.455823 | | 0.5112991 | | 0.0012394 | |
| **28** | Eicosapentaenoic acid | | 1.83389 | | 2.57068227 | | 0.00172142 | |
| **29** | Inosine | | 15.61506 | | 0.23790922 | | 0.00194531 | |
| **30** | Uridine | | 7.763475 | | 0.51910135 | | 0.00244185 | |
| **31** | Glutathione | | 1.719826 | | 0.17146603 | | 0.00254745 | |
| **32** | L-Threonate | | 3.013623 | | 10.0509136 | | 0.00261713 | |
| **33** | 2'-Deoxyuridine | | 5.305259 | | 0.15491428 | | 0.00269285 | |
| **34** | Palmitic acid | | 5.257892 | | 1.70888128 | | 0.00269902 | |
| **35** | 2'-O-methylguanosine | | 1.645845 | | 0.14386935 | | 0.00284149 | |
| **36** | Pantetheine | | 1.035304 | | 0.04784106 | | 0.00292276 | |
| **37** | L-Tyrosine | | 1.149014 | | 0.56714026 | | 0.00336316 | |
| **38** | L-Glutamate | | 6.92656 | | 1.61723038 | | 0.00414166 | |
| **39** | 2'-O-methylinosine | | 4.558741 | | 0.07774437 | | 0.00423794 | |
| **40** | L-Phenylalanine | | 1.69186 | | 0.30595588 | | 0.0043788 | |
| **41** | 3-Phospho-D-glycerate | | 3.372723 | | 0.24466538 | | 0.00471807 | |
| **42** | N-Acetylglucosamine 1-phosphate | | 1.881344 | | 0.10554396 | | 0.00505822 | |
| **43** | Thymidine | | 8.245629 | | 0.24547027 | | 0.00695409 | |
| **44** | L-Leucine | | 3.724594 | | 0.3953055 | | 0.00737488 | |
| **45** | (4Z,7Z,10Z,13Z,16Z,19Z)-4,7,10,13,1 6,19-Docosahexaenoic acid | | 2.413397 | | 7.03660358 | | 0.00766056 | |
| **46** | Glutathione disulfide | | 4.200221 | | 0.4654991 | | 0.00855429 | |
| **47** | 1-Methylpseudouridine | | 1.709868 | | 0.24397297 | | 0.00969525 | |
| **48** | L-Pyroglutamic acid | | 2.270712 | | 2.73793272 | | 0.0098022 | |
| **49** | ketoisocaproic acid | | 4.363313 | | 0.34159616 | | 0.00992942 | |
| **50** | Xanthosine | | 1.284678 | | 0.01386461 | | 0.01245193 | |
| **51** | 1-Palmitoyl-2-oleoyl-phosphatidylglycerol | | 2.916177 | | 3.19018311 | | 0.01556341 | |
| **52** | sn-Glycerol 3-phosphoethanolamine | | 1.163672 | | 2.67852077 | | 0.01572588 | |
| **53** | Cyclic adenosine diphosphate ribose | | 1.874558 | | 0.1929919 | | 0.01858036 | |
| **54** | cis-Aconitate | | 1.416691 | | 0.32097334 | | 0.01888864 | |
| **55** | Citraconic acid | | 1.104553 | | 0.33509491 | | 0.02166299 | |
| **56** | Guanosine 5'-monophosphate (GMP) | | 1.018514 | | 2.78113518 | | 0.02899415 | |
| **57** | gamma-L-Glutamyl-L-glutamic acid | | 1.850272 | | 3.24982871 | | 0.02929046 | |
| **58** | Glycerol 3-phosphate | | 1.320309 | | 0.18351405 | | 0.03093437 | |
| **59** | Prostaglandin E2 | | 1.623172 | | 3.68716868 | | 0.03494251 | |
| **60** | L-Aspartate | | 1.385579 | | 1.61181971 | | 0.04211752 | |
| **61** | Uracil | | 1.015065 | | 0.58438137 | | 0.04727463 | |

VIP, variable importance in the projection; FC, fold change.

FC was calculated as the ratio of the average variable value in the A549 group to that in the Beas-2B group.

[**Figure legends**](file:///D:\360downloads\baidu-translate-client\resources\app.asar\app.html)**:**

# Fig.S1. (A) UHPLC of resveratrol standard sample. (B)UHPLC of resveratrol dimer reaction sample. (C) Mass spectrum of TVN synthesized by horseradish peroxidase.

# Fig.S2. (A) Apoptotic A549 cells were assessed by Annexin V-FITC/PI fluorescent intensity at 60 h under indicated treatments. (B) Measurement of intracellular ROS production in A549 cells was using the fluorescent probe DCFH-DA via FlowJo 7.6 analysis. Red peak, cells without treatment; blue peak, after treatment with 10 μM TVN for 60 h. (C) The ΔΨm was detected by FCM via JC-1 staining.

# Fig.S3. Total ion chromatograms (TIC) of cells metabolites under positive (A) and negative (B) ion modes. Metabolomics showed very stable performance as chromatograms were anastomotic in positive ion and negative ion models.QC1-3 indicates n=3 test per mode.

**Fig.S4.** OPLS-DA analysis of LC/MS data from A549 verses Beas-2B **(A**, **B**, **C**, **D)** and A549D verses A549 **(E**, **F**, **G**, **H)** under positive and negative modes. Identified compounds could be separated well with optimal goodness of fit. **A**, **B**, **E**, **F**: OPLS-DA score plot. **C**, **D**, **G**, **H**: Validated model plots obtained by permutation test.

**Fig.S5.** **(A)**The clustering results of hierarchical cluster analysis from A549 group verses Beas-2B group based on the significantly diﬀerent metabolites under positive and negative modes. **(B)** Enriched KEGG pathway for the differential metabolites from A549 group verses Beas-2B group.

**Fig.S6.** **(A)** Volcano map of diﬀerentially expressed protein from A549 group verses Beas-2B group; **(B)** Volcano map of diﬀerentially expressed protein from A549D group verses A549 group.

**Fig.S7.** **(A)** Gene Ontology analysis for Biological processes (BP), molecular functions (MF), and cellular components (CC) of identified proteins based on significant diﬀerent proteins from A549 verses Beas-2B; **(B)**The first 10 pathways that contain the highest number of proteins and metabolites.
